# The *FRIABLE1* Gene Product Affects Cell Adhesion in Arabidopsis

**DOI:** 10.1371/journal.pone.0042914

**Published:** 2012-08-14

**Authors:** Lutz Neumetzler, Tania Humphrey, Shelley Lumba, Stephen Snyder, Trevor H. Yeats, Björn Usadel, Aleksandar Vasilevski, Jignasha Patel, Jocelyn K. C. Rose, Staffan Persson, Dario Bonetta

**Affiliations:** 1 Max Planck Institute of Molecular Plant Physiology, Golm/Potsdam, Germany; 2 Vineland Research and Innovation Centre, Vineland Station, Ontario, Canada; 3 Department of Cell and Systems Biology, University of Toronto, Toronto, Ontario, Canada; 4 Department of Plant Biology, Cornell University, Ithaca, New York, United States of America; 5 Faculty of Science, University of Ontario Institute of Technology, Oshawa, Ontario, Canada; Umeå Plant Science Centre, Sweden

## Abstract

Cell adhesion in plants is mediated predominantly by pectins, a group of complex cell wall associated polysaccharides. An *Arabidopsis* mutant, *friable1* (*frb1*), was identified through a screen of T-DNA insertion lines that exhibited defective cell adhesion. Interestingly, the *frb1* plants displayed both cell and organ dissociations and also ectopic defects in organ separation. The *FRB1* gene encodes a Golgi-localized, plant specific protein with only weak sequence similarities to known proteins (DUF246). Unlike other cell adhesion deficient mutants, *frb1* mutants do not have reduced levels of adhesion related cell wall polymers, such as pectins. Instead, FRB1 affects the abundance of galactose- and arabinose-containing oligosaccharides in the Golgi. Furthermore, *frb1* mutants displayed alteration in pectin methylesterification, cell wall associated extensins and xyloglucan microstructure. We propose that abnormal FRB1 action has pleiotropic consequences on wall architecture, affecting both the extensin and pectin matrices, with consequent changes to the biomechanical properties of the wall and middle lamella, thereby influencing cell-cell adhesion.

## Introduction

The middle lamella, which is formed during cell division, allows for cell-cell adhesion between plant cells. The principle component of the middle lamella is pectic polysaccharides (pectins; [1]). Pectins comprise three principle classes: variably esterified homopolymers of galacturonic acid (homogalacturonan; HG); polymers of alternating rhamnose and galacturonic acid residues that are substituted with arabinan and galactan sidechains, rhamnogalacturonan I (RGI), and rhamnogalacturonan II (RGII); a structural pectin consisting of a galacturonic acid backbone with complex saccharide side chains [2]. These polymers are present in differing ratios in both the primary cell wall, where they form a complex assemblage with the other major polysaccharides, such as cellulose and hemicelluloses, and in and middle lamella [3].

The most abundant pectic polysaccharides in the middle lamella are HGs with a low degree of methylesterification; a characteristic that may promote cell adhesion since pectin demethylation can enhance calcium cross-linking of adjacent HG chains and consequent gel formation [4]–[8]. HG-mediated cell adhesion is thought to require the action of apoplastic pectin methylesterases (PMEs) since HGs are often secreted in a highly esterified form [2].

The middle lamella also contains structural cell wall proteins, such as hydroxyproline-rich glycoproteins [9], [10], glycine-rich proteins [11] and arabinogalactan proteins (AGPs). The latter may be implicated in cell adhesion because of the apparent binding to pectins [1], [12]–[14]. Other possible modes of cell adhesion include cross-linking of polymers in middle lamella with components of the primary cell wall. For example, it is evident that some pectins are linked to xyloglucans (XyG) [15], [16], which in turn associate with cellulose microfibrils. Indeed, recent reports suggest that XyGs are involved in cell adhesion [17].

Several mutations that lead to reduced cell adhesion affect pectin-related functions [18]–[20]. For example, *quasimodo1* (*qua1*) shows a reduction in cell adhesion attributed to defective HG synthesis [18], [21]. A recombinant *QUA1*-related protein, GAUT1, can transfer GalA residues onto HG oligomers [22], further indicating that QUA1 plays a role in HG synthesis. Plants carrying mutations at the *QUA2/TSD2* locus also show reductions in both cell adhesion [19], [23], and HG content [19]. The corresponding gene encodes a Golgi-localized membrane protein that contains a predicted methyltransferase domain [19], [23]. This suggests that HG synthesis and cell adhesion require both polymerase and methyltransferase activities [19]. Other genes that have been implicated in pectin synthesis by virtue of their cell adhesion defects, and their homology to glycosyltransferases, are *EPC1* [20] and, in tobacco, *NpGUT1* [24]. The latter encodes a putative glycosyltransferase that was thought to be involved in RGII synthesis [24]. However, this contention has recently been disputed following the characterization of loss-of-function mutations in two closely related genes, *IRX10* and *IRX10-L* genes [25], [26] that are deficient in xylan.

Another type of cell adhesion deficiency is perturbed cell separation, or organ fusion, which can happen as a consequence of defective cuticular wax formation (for review see [27], [28]). One explanation for the ectopic fusions is that the cuticle normally blocks cell wall interactions between adjacent organs, which prevent ectopic adhesion. However, the variation in developmental phenotypes among wax mutants suggests that other unknown mechanisms also are involved in establishing these fusions [28].

Here, we report the identification and characterization of an *Arabidopsis* gene, *FRIABLE1* (*FRB1*), which affects both cell adhesion and organ fusion. *FRB1* encodes a Golgi localized, plant specific, membrane protein with weak similarity to known proteins, and appears to be required for cell wall integrity.

## Results

### *frb1* seedlings display both cell dissociation and adhesion phenotypes

**Figure 1:**
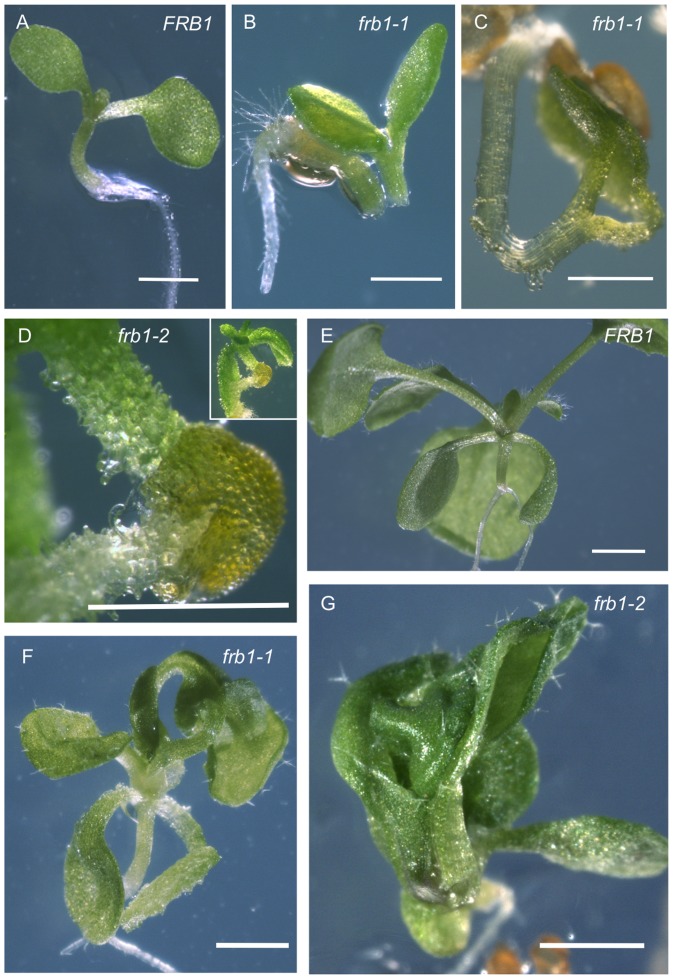
Phenotypic variation in *frb1* mutant seedlings. A. Wild-type *FRB1* seedling after 5 days growth on MS media. B. A 3-day old *frb1–1* seedling where the hypocotyl has spontaneously broken during germination. C. An example of a *frb1–1* seedling where the cotyledons have become fused as the seedling germinated. D. A *frb1–2* seedling showing severe cell dissociation. E. A two week old *FRB1* seedling grown on MS media. F. and G. At the same stage of growth as seedlings in E, *frb1–1* and *frb1–2* seedlings have little cell separation but many do have extensive fusions between leaves and other aerial organs. All scale bars equal 0.5. mm.

To identify *Arabidopsis* mutants with cell adhesion defects we conducted a visual screen on approximately 10,000 seedlings from a segregating T2 population transformed with a pCAMBIA1300 derivative (CAMBIA, Black Mountain, Australia). Although a number of seedlings with aberrant morphologies were identified, one mutant had an obvious fused cotyledon phenotype which we could easily identify using a dissecting microscope. We later confirmed that this was a recessively segregating mutant, which we named *friable1* (*frb1*). The *frb1* seedlings displayed three interrelated phenotypes: cell dissociation, spontaneous breakage, and ectopic organ fusion (Figure 1). The cell dissociation phenotype involved the sloughing of cells in seedlings to the point where tissues appeared to crumble, or were “friable” (Figures 1C and D). In other cases (10% of the time), dissociation of entire plant parts occurred, leading to spontaneous organ breakage (Figure 1B). Seedlings that showed severe cell dissociation typically died. Cell dissociation defects were not apparent in roots or in adult plants (data not shown). Paradoxically, *frb1* plants also displayed a defective ectopic organ fusion phenotype. In many instances (40% of the time), seedlings with fused cotyledons (Figure 1C), or fused hypocotyls and cotyledons (Figures 1F and G), were observed. These contrasting phenotypes suggest that FRB1 is required for cell adhesion in seedling tissues.

### Loss of *FRB1* function affects embryo development and leads to abundant surface homogalacturonan in seedlings

**Figure 2:**
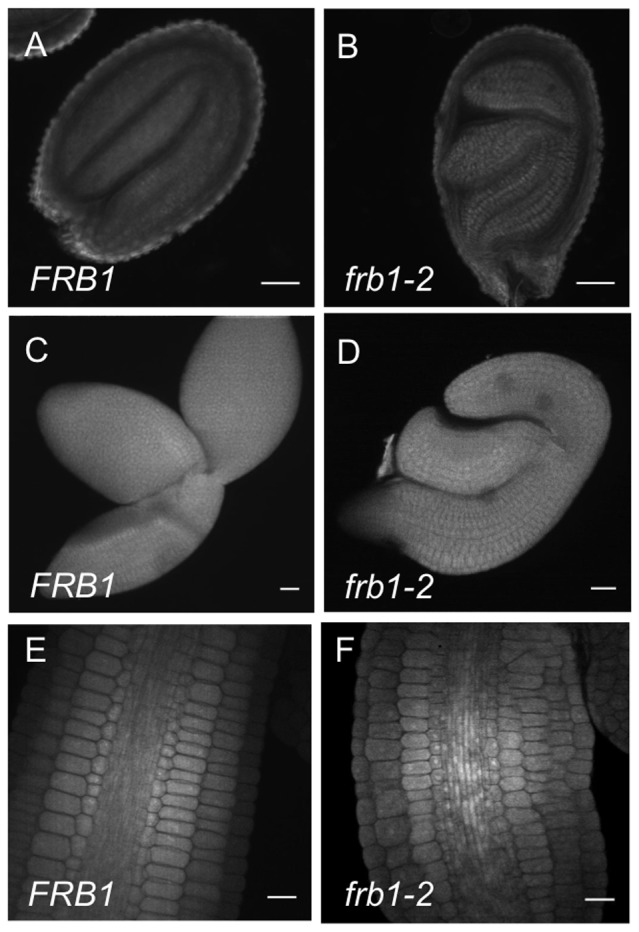
Comparison of embryo morphology of *FRB1* and *frb1–1.* A. Optical section through a *FRB1* seed. B. Optical section through a *frb1–1* seed. C. *FRB1* embryo that has been dissected out of the seed coat. D. *frb1–1* embryo that has been dissected out of the seed coat. E. Optical section through hypocotyl of a *FRB1* embryo where tissue layers are visible. F. Optical section through hypocotyl of a *frb1–1* embryo showing partly disrupted cell files. Scale bars are 20 µm.

The defects in cell adhesion were also observed in *frb1* embryos (Figures 2A to F), which displayed distorted morphology where cotyledons did not expand fully and, instead of flattened discs as in wild-type, were cup-shaped (Figures 2A to D). There were no apparent differences in tissue organization in *frb1* compared to *FRB1*, although cells in frb1 were more variable in size and shape as a result of uneven cell files (Figures 2E and F). These phenotypes might be due to poor cell adherence allowing cells to slide past one another during embryo development.

**Figure 3:**
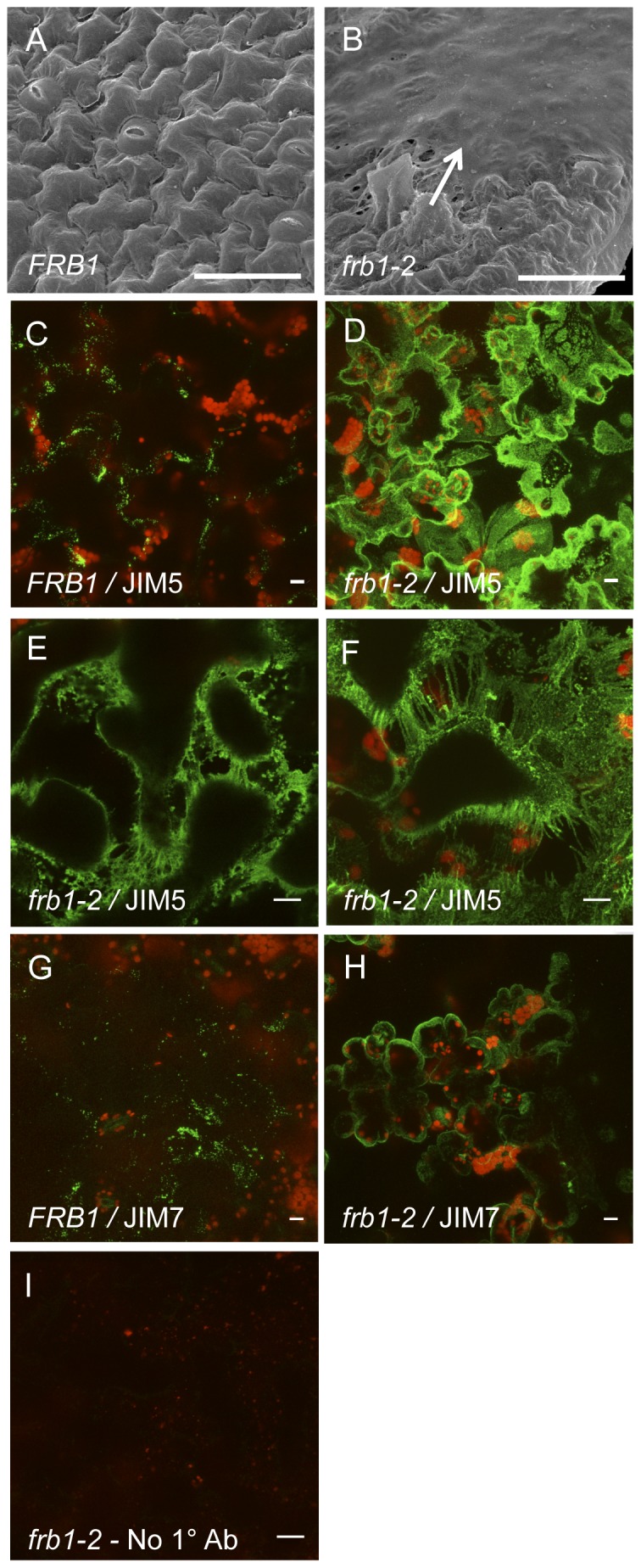
Surfaces in *frb1* cotyledons are coated in pectins. A. Electron micrograph of epidermal cell surface of a *FRB1* cotyledon. B. Electron micrograph of epidermal cell surface of an *frb1–2* cotyledon occluded by pectins (arrow). C. Whole mount an *FRB1* cotyledon showing JIM5 dependent signal (green). Red color is from chlorophyll autoflourescence. D–F. Whole mount an *frb1–2* cotyledons showing JIM5 dependent signal. JIM5 epitope is present on both the surface and in between *frb1* cells. G. Whole mount an *FRB1* cotyledon showing JIM7 dependent signal (green). H. Whole mount an *frb1–2* cotyledons showing JIM7 dependent signal. Scale bars equal 10 µm.

Using scanning electron microscopy we observed a substance exuding from *frb1* cotyledon cells (Figure 3). To determine if this substance consisted of pectic polymers, we used the antibodies JIM5 and JIM7 to assess the presence of HG epitopes using whole seedlings without chemical fixation to limit antibody access to cell surfaces. While the surfaces of wild-type cotyledons were sparsely decorated by JIM5 (Figure 3C), those of *frb1* were uniformly decorated (Figures 3D to F). Separated cells displayed long JIM5 cross-reactive strands connecting the cells (Figures 3E and F). Similarly, JIM7 epitopes were also more extensively stained in *frb1* plants (Figures 3G and H). This indicates that at least a portion of the surface substance is HG.

### Cloning and characterization of *FRB1*

To determine the location of the T-DNA insertion in *frb1–1* we conducted TAIL-PCR (Liu *et al.* 1995). The T-DNA insertion in *frb1–1* was located to the upstream region of At5g01100 (Figure S1A). We confirmed that this locus corresponded to the *frb1* mutant phenotype by analyzing two additional T-DNA insertion mutants (SALK_078459; *frb1–2*, and WiscDsLox1D5; *frb1–3*) that have inserts at the *FRB1* gene locus. We further confirmed that these alleles held reduced *FRB1* transcript by RT-PCR as no transcript was detected after 30 PCR cycles (Figure S1B). However, when we increased the number of PCR cycles to 40, we detected a faint band in *frb1–1* (Figure S1B), suggesting that while *frb1–2* and *frb1–3* are most likely mRNA nulls, *frb1–1* is only a partial loss-of-function. This result is consistent with the location of the T-DNA insertion in *frb1–1* (in the predicted promoter region).

The *FRB1* gene is predicted to encode a 631-amino acid protein (71.3 kD) with a putative N-terminal transmembrane domain (TMHMM Server v. 2.0; CBS, Denmark), and to be a type II transmembrane protein. It has a conserved plant-specific domain of unknown function (DUF246) spanning about 300 amino acids in the C-terminal region (Figure S1C). The protein does not contain any other unambiguous motifs that would suggest a possible function. In *Arabidopsis* at least 34 predicted proteins occur with significant homology to FRB1 (Figure S2). The conserved DUF246 domain is found in at least 210 proteins in different plant species (InterPro number: IPR004348), including 71 in *Arabidopsis*.

### Expression of *FRB1*

**Figure 4:**
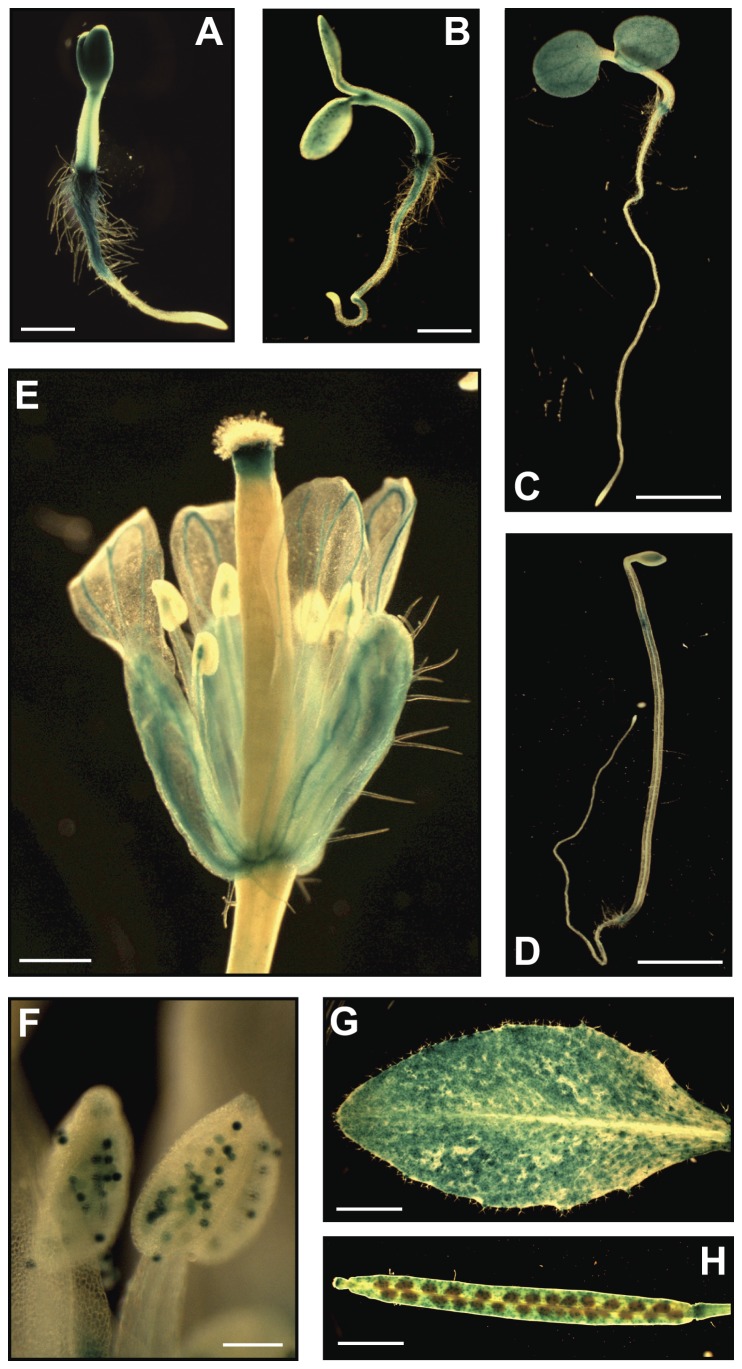
Expression of the *FRB1* gene. A to H. The FRB1 expression was examined using the 4.3 kbp promoter sequence just upstream of the FRB1 start codon fused to GUS. A and B. GUS activity in two and three-day-old seedlings, respectively. Scale bars equal 1 mm. C. GUS activity in five-day-old seedlings. Scale bar equals 5 mm. D. GUS activity in five-day-old etiolated seedlings. Insert shows blow-up of the expansion zone. Scale bar equals 5 mm. E. GUS activity in flowers. Scale bar equals 1 mm. F. GUS activity in anthers. Scale bar equals 100 µm. G. GUS activity in rosette leaves. Scale bar equals 5 mm. H. GUS activity in siliques. Scale bar equals 4 mm.

To examine *FRB1* gene expression we generated transgenic plants containing a β-glucuronidase (GUS) reporter gene under control of the complete intergenic region (4.3 kbp) between the ATG start codon for *FRB1*, and the stop codon for the upstream gene. GUS activity was weak in germinating seeds (Figure S3A), but became stronger during early seedling development (Figures 4A and B; S3B to E), particularly at the junction between hypocotyl and root, in emerging cotyledons, and in parts of the roots in 2-day-old seedlings (Figures 4A and S3B). The *FRB1* promoter was less active in older seedlings (Figure 4C). The strong activity of the *FRB1* promoter in young seedlings is consistent with the observed mutant phenotypes. However, the *FRB1* promoter was also active in the inflorescence (sepals, petals, mature pollen, and siliques) and rosette leaves (Figures 4E to H).

### FRB1 is localized to the Golgi apparatus

**Figure 5:**
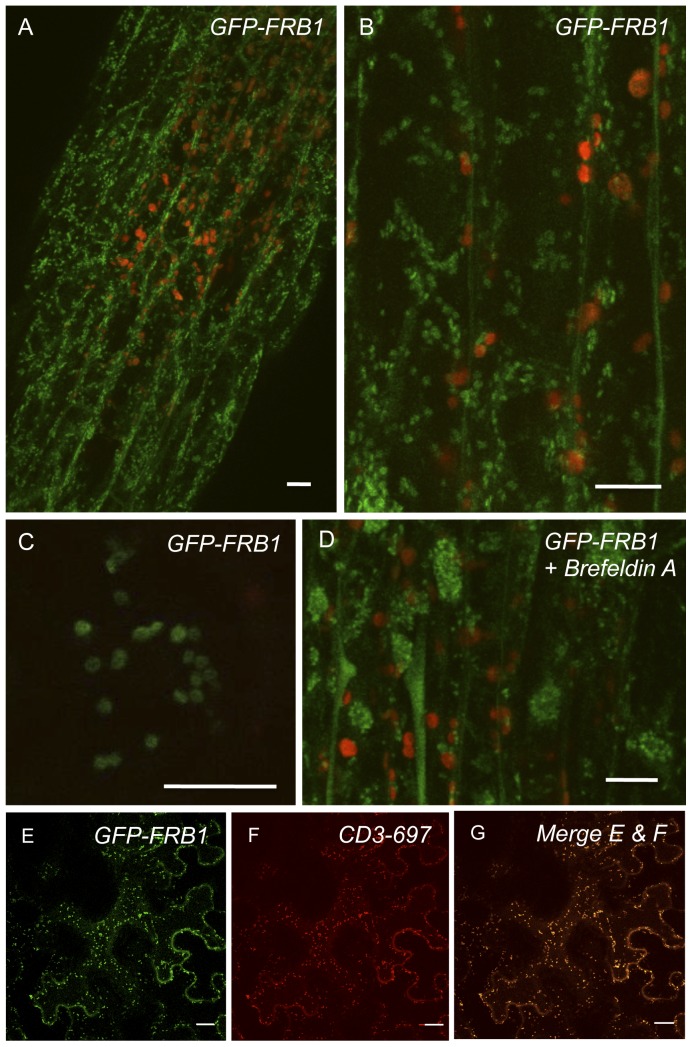
GFP-FRB1 fusion protein accumulates in subcellular compartments. A. Hypocotyl cells of transgenic plants expressing GFP-FRB1 fusion protein under the control of a constitutive promoter. Note that the GFP accumulates in subcellular compartments (green). Red fluorescence is from chloroplasts. B. GFP-FRB1 fluorescent particles in the cortical cytoplasm. C. Higher magnification image showing the ring morphology of the GFP-tagged compartments. D. Hypocotyl cells of GFP-FRB1 plants treated with 100 µg/ml Brefeldin A for 15 min. Note the redistribution of the GFP-labeled compartments to aggregates, especially around nuclei. Some of the GFP signal has also become soluble. E. Transient over-expression of GFP-FRB1 fusion protein or F. mCherry (CD3–967) fusion protein in an epidermal tobacco cell. G. Micrograph showing overlap of E and F. Scale bars equal 10 µm in A–D and 20 µm in E–G

To investigate the sub-cellular localization of FRB1 we generated an N-terminal translational fusion of GFP (green fluorescent protein) to FRB1 (GFP-FRB1), which complemented the *frb1* mutant phenotypes (Figure S4). The GFP signal in transgenic GFP-FRB1 Arabidopsis plants was present in small, highly motile (Movie S1), compartments in the cortical cytoplasm, which traveled within cytoplasmic strands, similar to previously reported Golgi-localized proteins ([29] Figures 5A to C). The compartments were affected by inhibitors that target Golgi dynamics [29], [30], such as the actin inhibitor Latrunculin B (1 µM; 15 min of treatment; Movie S2), and the protein transport inhibitor Brefeldin A (BFA) (Movie S3), which inhibits protein trafficking from the endoplasmic reticulum (ER) to the Golgi apparatus ([31]; Figure 5D). Treatment of GFP-FRB1 with BFA resulted in concentration of large perinuclear aggregates, consistent with previously identified “Brefeldin A compartments” [30], [32]. Furthermore, transient co-expression of GFP-FRB1 and a Golgi-marker 9(CD3–967) [33] in tobacco cells revealed overlapping localization (Figures 5E to G). In addition, western blots of the Golgi-tagged FRB1 protein indicates that it is localized to a membrane fraction (Figure S5). These results indicate that FRB1 is likely localized to a compartment similar to the Golgi apparatus.

### *frb1* mutants display complex cell wall alterations

**Figure 6:**
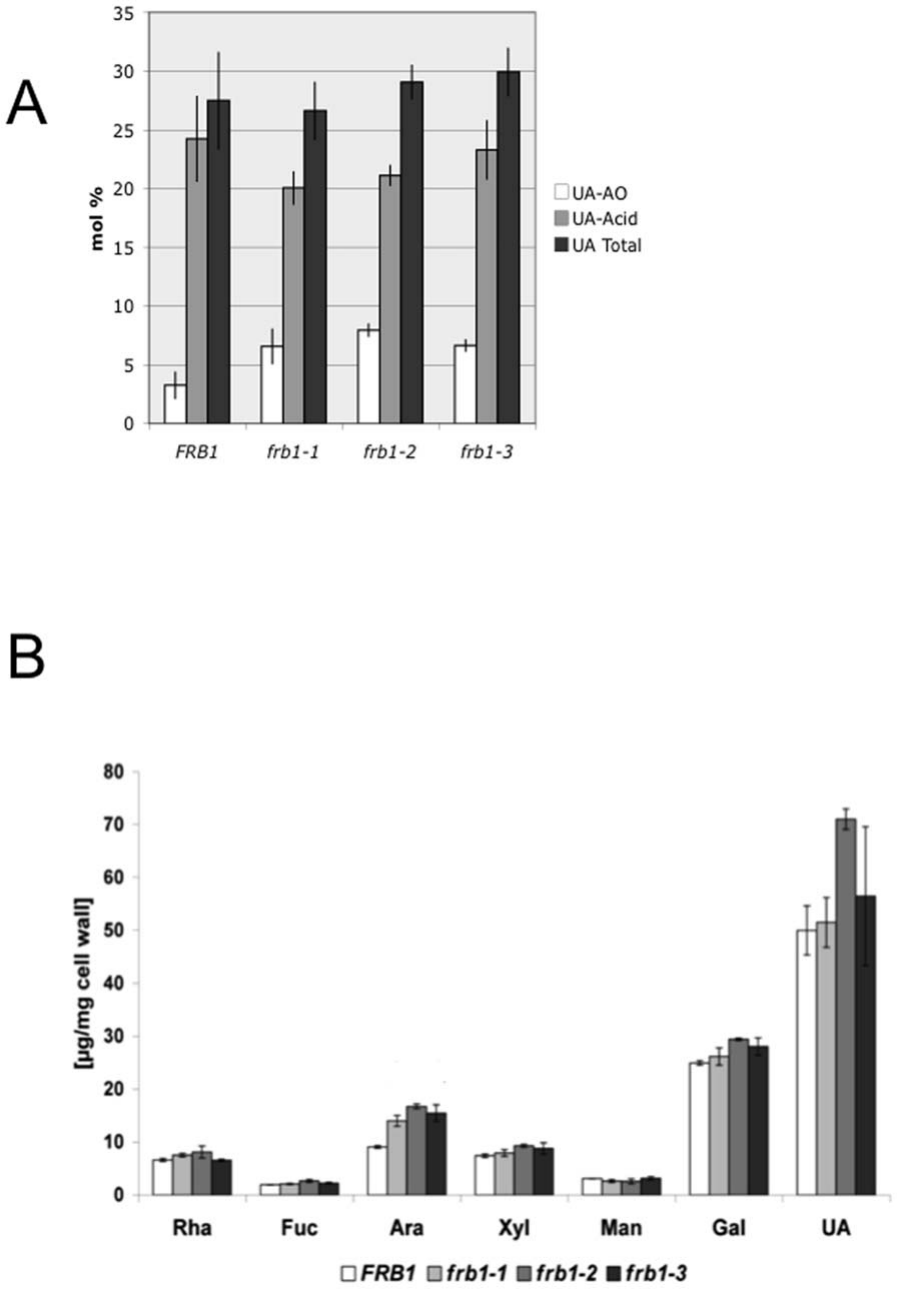
Monosaccharide content of *FRB1* and *frb1* seedlings. A. Uronic acid levels in ammonium oxalate (AO) soluble fraction and in acid soluble (1 M H_2_SO_4_, UA-Acid) fraction. Levels for AO soluble fraction are significantly different with p<0.05 for all three *frb1* lines compared to wild-type B. Neutral sugar content of extracted cell walls from *FRB1* and *frb1* seedlings. Abbreviations: Rha, rhamnose; Fuc, fucose; Ara, arabinose; Xyl, xylose; Gal, galactose; Glc, glucose. Error bars represent standard deviations with a sample size of 4 and 3 for Figures A and B, respectively. Levels of arabinose are significantly different with p<0.05 for all three *frb1* lines compared to wild-type

**Figure 7:**
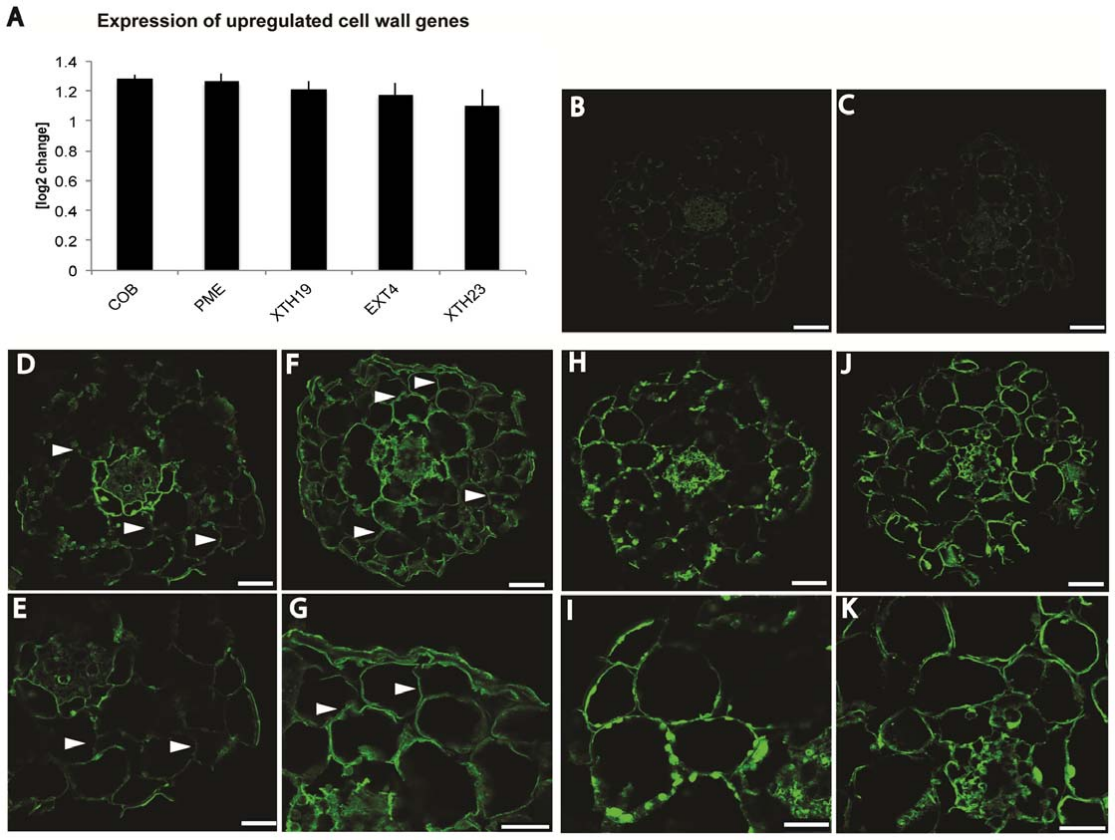
Annotation of cell wall genes upregulated in *frb1* and immunolabeling of transverse hypocotyl sections using antibodies against arabinosylated cell wall epitopes. A. Differentially expressed genes involved in cell wall metabolism. Increase of expression of in *frb1* upregulated cell wall genes with n = 3. Error bars are ± standard deviation. B. to K. Immunolabeling of transverse hypocotyl sections (10 µm) using antibodies against arabinosyl epitopes predominantly present in extensins (LM1; D to G) or in RGI side chains (LM6; H to K). B. *FRB1* anti-rat control. C. *frb1–1* anti-rat control. D. and E. *FRB1* staining using LM1. F. and G. *frb1–1* staining using LM1. H. and I. *FRB1* staining using LM6. J. and K. *frb1–1* staining using LM6. Scale bars in B, C, D, F, H, J equal 50 µm, scale bars in E, G, I, K equal 25 µm.

To determine whether the *frb1* phenotypes are associated with changes in cell wall composition, we first assessed whether the observed differences in antibody staining of whole *frb1* seedlings were a result of increased accessibility or abundance of HG polymers. We therefore measured the release of uronic acids from *frb1* mutants using different extraction methods (Figure 6A; Table 1). We first treated ten day-old seedlings gently with ammonium oxalate, which extracts a limited portion of cell wall pectins [34], [35]. This treatment solubilized a higher proportion of uronic acids from *frb1* seedlings than from *FRB1* (Figure 6A), but this difference was no longer apparent when the remaining uronic acids were extracted using hot acid (Figures 6A and B). This suggests that certain uronic acids were more accessible or differentially modified in *frb1*.To establish whether *frb1* also has altered cell wall sugar composition, we measured the monosaccharides in the *frb1* mutants (Figure 6B). This revealed that the arabinose content was 50–80% (5–7 µg/mg cell wall) higher in *frb1* as compared to wild-type (Figure 6B). However, no changes were observed in the absolute uronic acid content (Figures 6A and B; Table 1). Arabinose is a major component of pectin side chains and of the glycosyl moieties of arabinogalactan proteins (AGPs) and extensins. To investigate whether the changed arabinose content is associated with a specific polymer type we fractionated cell wall material to enrich for certain matrix polymers. Neutral sugar analysis of the sequential extracts revealed that the increase of arabinose is consistent among all fractions (20–30 % in the CDTA and Na_2_CO_3_ fractions, and 40–80% in the 4 M KOH and the insoluble fractions; Table 1). We reasoned that the increased arabinose levels associated with the pectic fractions may signify higher levels of RGI-holding arabinan portions. To test this we used the LM6 monoclonal antibody, which binds α-(1–5)-L-arabinans [36], [37], which is predominately found in RGI side chains, but LM6 may also bind to some AGPs (http://www.plantprobes.net). We also tested whether we could detect differential binding of LM1 antibody, which binds preferentially to the glycan component of extensins [9]. We observed slightly increased LM1 labeling, particularly in the cortical region of mutant hypocotyls, but LM6 patterning in *frb1* sections was indistinguishable from that seen in *FRB1* (Figures 7D to K). Although no major changes were detected in galactose from the crude cell wall analysis, we observed differences in fractionated cell wall material (Table 1). Interestingly, galactose abundance was lower (7%) in the pectic CDTA fraction, but greater (approximately 15% to 40%) in the 4 M KOH and insoluble fractions, respectively (Table 1). These data suggest that either the incorporation of galactose and arabinose into certain polymers is abnormal in the mutants or that structural alterations promote extractability.

**Table 1:** composition after fractionation of cell wall material derived from 10 day old shoots of light grown seedlings.

		Rhamnose		Fucose		Arabinose		Xylose		Mannose		Galactose		UA [ µg/mg]	
		AVG	SD	AVG	SD	AVG	SD	AVG	SD	AVG	SD	AVG	SD	AVG	SD
**CDTA**	***FRB1***	12.2	0.5	1.9	0.0	20.1	0.6	5.2	0.4	3.3	0.7	57.3	1.3	47.1	7.6
	***frb1-1***	12.3	1.4	1.6	0.4	**25.7**	0.9	5.5	0.3	3.7	0.2	**51.1**	1.7	42.0	4.3
	***frb1-2***	11.2	1.6	1.5	0.7	**24.3**	3.1	5.4	0.2	3.2	0.3	**54.4**	3.4	40.8	12.6
	***frb1-3***	11.4	0.3	**1.7**	0.0	**24.7**	0.4	5.4	0.1	3.0	0.2	**54.0**	0.8	42.6	3.8
**Na_2_CO_3_**	***FRB1***	18.9	0.3	2.8	0.5	23.9	0.8	6.8	0.6	10.6	0.2	37.0	0.8	29.3	8.1
	***frb1-1***	**14.8**	0.4	2.5	0.2	**32.1**	0.5	6.8	0.3	**8.2**	0.3	35.6	0.6	20.6	9.4
	***frb1-2***	**15.7**	0.3	2.9	0.1	**31.7**	1.0	6.8	0.6	**7.0**	0.6	35.9	0.6	28.5	4.9
	***frb1-3***	**12.7**	0.3	2.3	0.2	**29.7**	0.3	7.1	0.2	**7.9**	0.2	**40.3**	0.5	25.4	3.6
**KOH**	***FRB1***	6.8	1.3	6.7	1.6	9.3	1.1	48.7	5.7	6.6	1.1	22.0	1.0		
	***frb1-1***	6.1	0.4	5.4	1.5	**15.4**	0.6	41.9	2.8	6.0	0.4	**25.3**	0.8		
	***frb1-2***	5.1	0.5	4.8	0.5	**13.3**	0.6	45.8	0.9	5.0	0.2	**26.0**	0.5	**cellulose [ µg/mg]**	
	***frb1-3***	4.9	1.6	**2.6**	1.8	**13.5**	1.4	41.0	1.1	7.7	0.3	**30.2**	1.8	AVG	SD
**INSBL**	***FRB1***	6.0	1.5	3.0	0.1	10.7	0.7	37.2	3.8	22.7	2.2	20.4	0.7	23.2	1.8
	***frb1-1***	7.8	0.8	**2.6**	0.1	**19.6**	1.8	**22.9**	2.0	**18.4**	0.8	**28.6**	0.4	21.3	2.2
	***frb1-2***	**1.1**	1.3	1.9	2.1	**23.0**	1.9	**29.8**	0.8	**16.0**	1.2	**28.3**	3.0	21.7	2.5
	***frb1-3***	**8.4**	0.9	**2.2**	0.2	**18.2**	0.3	**16.6**	0.9	22.1	0.3	**32.5**	0.4	19.3	0.5

Neutral sugars are expressed in mol%. Uronic acids and cellulose are expressed in [ µg/mg] fractionated cell wall material. Cellulose was measured after fractionation as release of hexoses from the pellet by Seaman hydrolysis. Significant alterations (n = 3; *: p<0.05, **: p<0.01) are bold and underlined.

Neutral sugars in mol %; significant alterations (p<0.05) are bold and underlined ; INSBL: insoluble, AVG: average, SD: standard deviation.

### *frb1* alters the galactose and arabinose ratio

**Figure 8:**
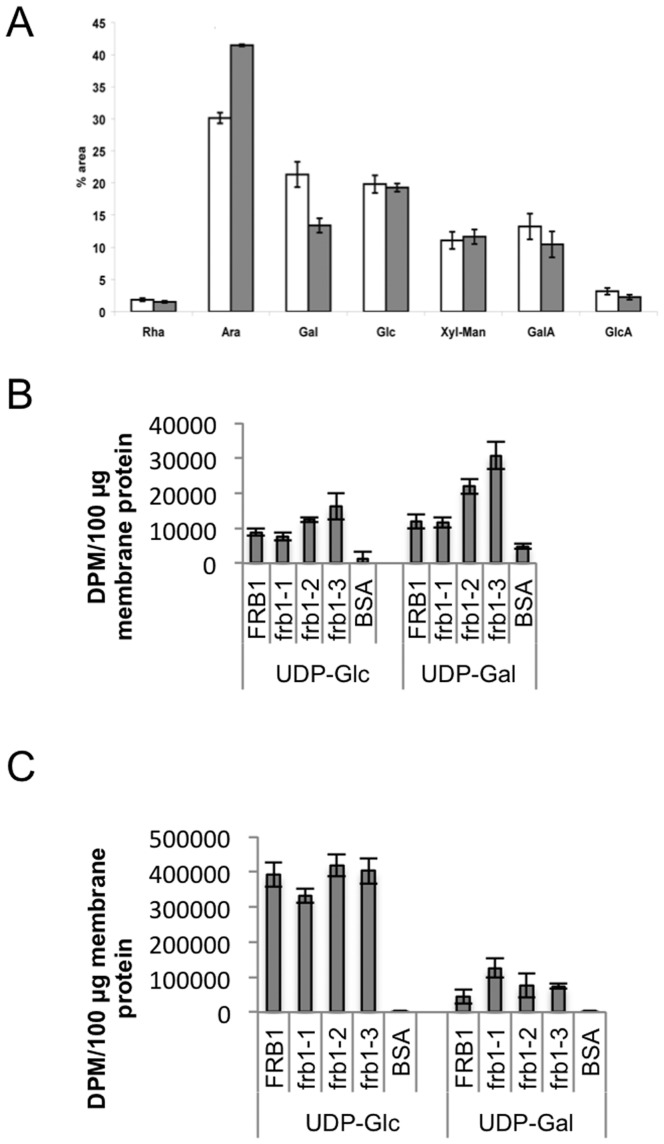
Sugar composition and sugar incorporation in membrane preparations from *FRB1* and *frb1* plants. A. Sugar composition derived from microsomal preparations of wild-type and *frb1*–*2*. Monosaccharides and uronic acids were identified based on retention time and comparison to pure standards and values are expressed in % of total area. Significant changes (two-tailed Student's t-test, n = 3, *: p<0.05; **: p<0.01) are marked with stars. Rha: rhamnose, Ara: arabinose, Gal: galacatose, Glc: glucose, Xyl-Man: unseparated peak containing xylose and mannose, GalA: galacturonic acid, GlcA: glucuronic acid. B. The total radioactivity in the chloroform insoluble material. C. The total radioactivity in the chloroform soluble material. See the experimental procedures for details.

To test whether the observed cell wall alterations originated from either biosynthetic, i.e Golgi-localized, or cell wall modifying reactions, i.e. apoplastic, we conducted a sugar analysis of microsomal membranes. This experiment revealed that the increased arabinosyl- and decreased galactosyl residues are already apparent in membrane compartments (Figure 8A; Table 2), and are thus likely due to enzyme activities in a membranous compartment. This is consistent with the membrane localization of the FRB1 protein, and could indicate that the efficiency of galactose and arabinose incorporation is altered due to FRB1 deficiency. In this case, we would expect to see lower incorporation of galactose in *frb1* membrane-enriched fractions. To test this we performed incorporation assays using membrane preparations from the *frb1* mutants and wild-type. Interestingly, the *frb1-der*ivedmembranes incorporated as much, or more, UDP-galactose as the wild-type fractions (Figures 8B and C), which was evident in both the protein and lipid fraction of the membranes (Figures 8B and C). These data indicate that *frb1* plants can utilize UDP-galactose but they incorporate less into galactose-harboring polymers such as XyGs, RGIs or glycoproteins.

Since FRB1 appears to be a Type II membrane protein, it could represent a glycosyltransferase. To test this we expressed FRB1 in insect cells and assayed it for activity using a range of substrates and targets (Table S1). None of the combinations tested resulted in incorporation of substrates (data not shown). While this does not exclude that FRB1 is a glycosyltransferase, the target substrate has yet to be identified.

**Table 2:** Neutral sugar composition derived from microsomal preparations of FRB1 and frb1-2 measured via alditol acetates and subsequent GC-MS analysis.

	Rha		Ara		Gal		Glc		Xyl		Man	
	mean	± SD	mean	± SD	mean	± SD	mean	± SD	mean	± SD	mean	± SD
***FRB1***	5.7	0.3	44.7	3.2	22.2	3	17.2	1.2	4.3	0.8	5.8	0.5
***Frb1-2***	**7.4**	0.4	**55**	0.5	**13.2**	1.2	14.9	0.8	**6.9**	0.2	**2.6**	0.3
t-test	0.005		0.029		0.024		0.056		0.023		0.003	

Monosaccharides are expressed in mol %. Significant changes (two-tailed Student's t-test, n = 3, *: p<0.05, **: p<0.01) are marked in bold and underlined. Rha: rhamnose, Ara: arabinose, Gal: galactose, Glc: glucose, Xyl: xylose, Man: mannose.

Mol% derived from GC-MS; bold and underlined are significant (Student's t-test); n = 3, p<0.05.

### *frb1* has modified xyloglucan structures

**Figure 9:**
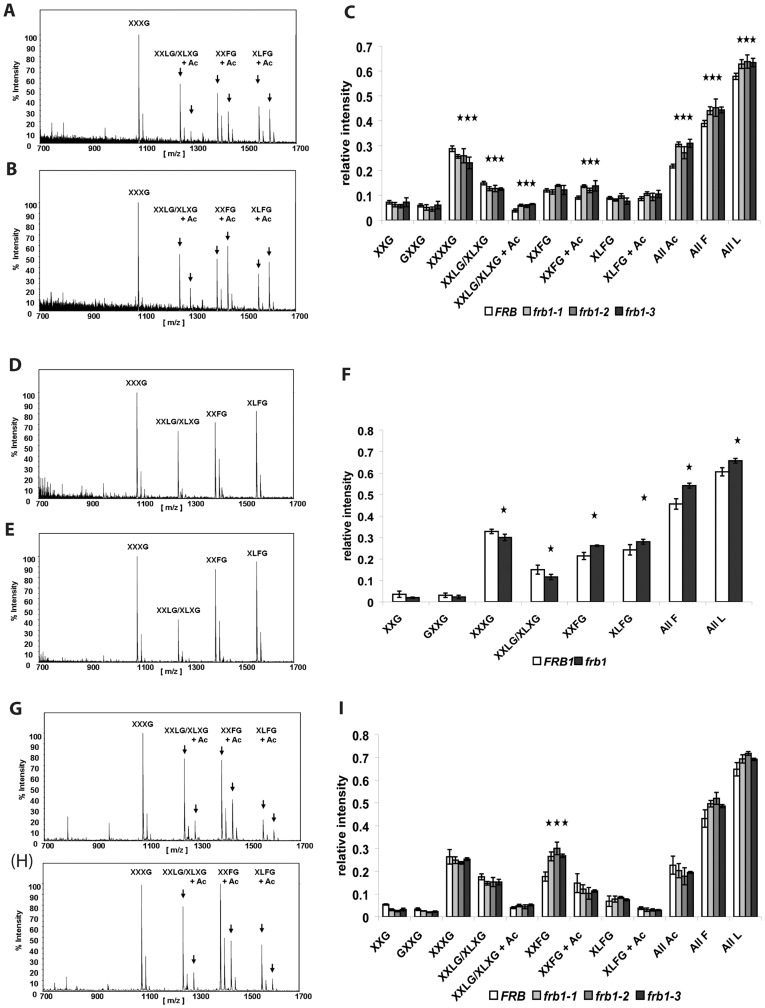
Xyloglucan oligosaccharide mass profile (OLIMP) of crude cell wall material from shoots (A, B, C) and its respective 4 M KOH fractions (D, E, F). A. and D. Representative spectra derived from *FRB1*. B. and E. Representative spectra derived from *frb1*. C. OLIMP analysis of shoots. D. OLIMP analysis of 4 M KOH fraction derived from shoots. Nomenclature of xyloglucan oligosaccharides are taken from [38] and references within. Stars indicate significant alterations with p<0.05.

The increase in galactose associated with the 4 M KOH fraction may signify an alteration in the hemicellulosic polymer XyG. The fine structure of XyG was therefore determined using oligosaccharide mass profiling (OLIMP) [38], [80]. This revealed increased abundance of galactose-containing XyG subunits (All L, Figures 9D to F) in the XyG-enriched 4 M KOH extract of *frb1*. Similarly, the relative amounts of galactosylated (All L), fucosylated (All F) and O-acetylated (All Ac) XyG oligosaccharides were all elevated in crude cell walls of *frb1* (Figures 9A to C). This was mainly due to a relative increase of one particular fucose- and galactose-containing oligosaccharide (XXFG+Ac). Neutral sugar analyses (Table 1) showed a net-reduction of galactose in the CDTA fraction, and a net-increase in the 4 M KOH fraction. Although OLIMP is not a quantitative method together with the fractionated sugar analysis the results suggest that there is a net-increase of galactosylation in XyG (Figures 9A to C) and a net-decrease of galactose-containing polymers in the CDTA fraction most likely originating from RGI or perhaps glycosylated cell wall proteins.

### Loss of FRB1 function leads to an up-regulation of genes relevant for cell wall integrity and desiccation protection responses

To gain more insight into processes that are affected by the *frb1* mutation we performed transcript profiling using RNA isolated from 10-day-old *frb1–1* and *FRB1* seedlings, and probed ATH1 microarrays in triplicates. The complete list of genes with altered expression levels in *frb1* compared to wild type is shown in Table S2. In total, 114 genes had an average fold-change of at least two in the *frb1–1* background. Eighty-seven of these were up-regulated, and 27 genes down-regulated. Many of the up-regulated genes are annotated as storage proteins (Table S4), which often are associated with late seed development, and desiccation tolerance [39]. Such genes are perhaps up-regulated to mitigate increased water loss that may occur due to disrupted tissue integrity in *frb1–1* seedlings [40], [41].

**Figure 10:**
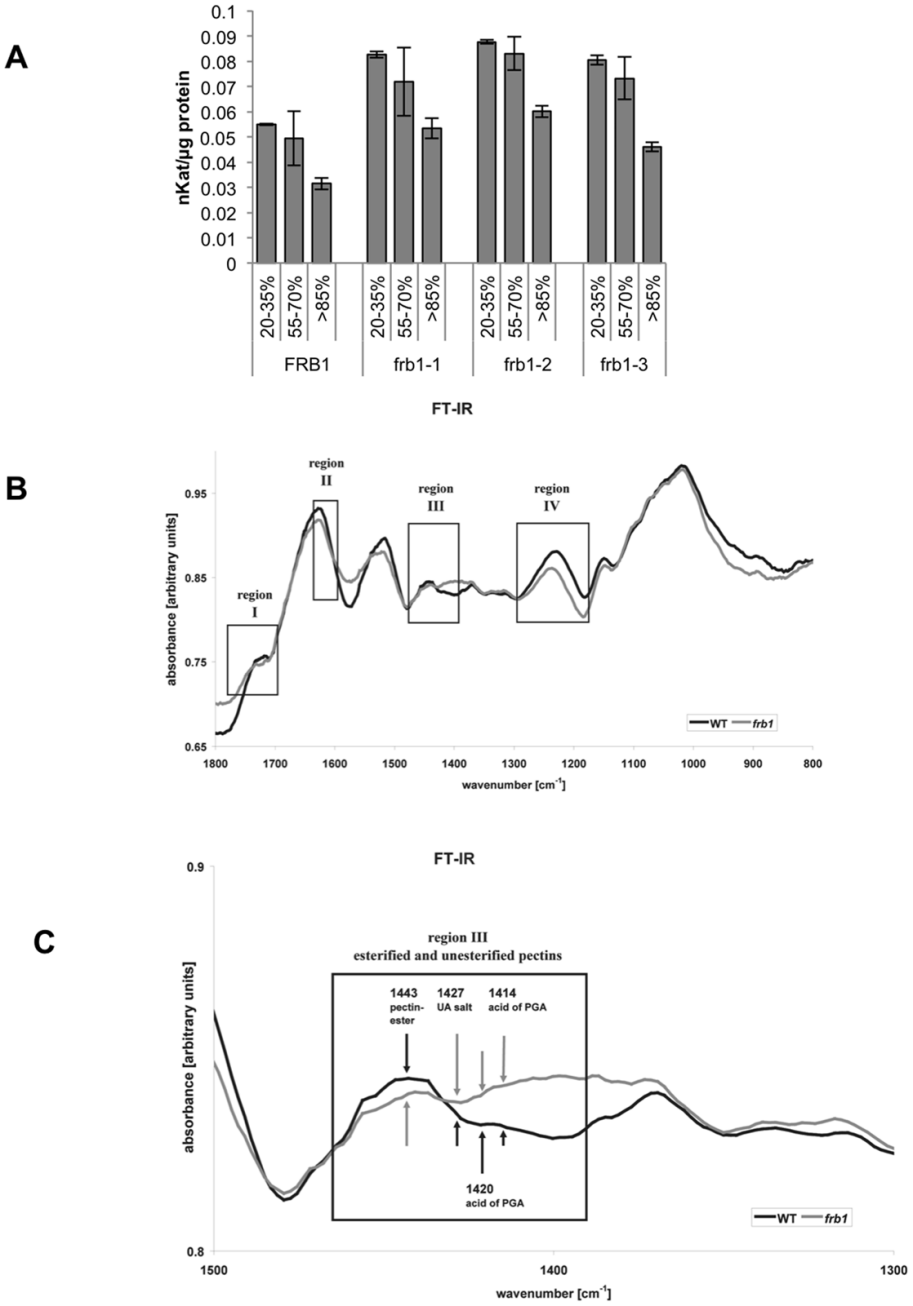
Comparison of pectin methylesterase (PME) activity in *FRB1* and *frb1* alleles and analysis of esterified and non-esterified cell wall regions using FTIR. A. PME activity in *frb1* alleles is from 45–70% higher than in wild-type seedlings. Degree of methylesterification of the pectin used as substrate in PME assays is indicated as percentage. Each column is the average of three separate assays; error bars indicate ± standard deviation. All levels are significantly different with p<0.05 for all three *frb1* lines compared to wild-type B. Comparison of *FRB1* and *frb1* of FTIR scans. C. Close-up of region III from A. showing particular differences in the degree of methylesterification between *FRB1* and *frb1*.

Interestingly, five cell wall-related genes, which may be related to cell adhesion/dissociation, were up-regulated (Figure 7A). In addition, all of these genes, namely a pectin-methylesterase (*PME*, At4g02330), the hydroxyproline-rich glycoprotein *EXTENSIN4* (*EXT4*, At1g76930), two XyG endo-transglucosylases/hydrolyases *XTH19* (At4g30290) and *XTH23* (At4g25810), as well as *COBRA* (At5g60920), encode presumed apoplastic structural or cell wall modifying proteins (Figure 7A). While up-regulation of *XTH19* and *XTH23* (previously termed *XTR6*) may be associated to differences in *frb1* XyG structure, we find it unlikely that these enzymes would impact on cell adhesion. Modifications in expression of cellulose-related genes, including *COBRA*, may indirectly affect XyG structures and pectin content [42]. However, no cell adhesion phenotype was reported when *COBRA* was over-expressed [43]. In contrast, the up-regulated PME gene may affect methylesterification and hence cell adhesion. Indeed, we observed increased *PME* activity in *frb1* seedlings (Figure 10A), suggesting that the *frb1* phenotypes may, at least in part, be caused by decreased pectin esterification [44]. We also performed FTIR analyses of the crude *frb1* cell wall material, which revealed changes in wave numbers that typically correspond to esterified pectins (Figures 10B and C). These include three regions [45], [46]; region I, which is related to esterified pectins; region II, which mainly accounts for pectates of un- or de-esterified pectins; and region III hosting signals for esterified pectins (1443), as well as for de-esterified pectins (1427, 1420, 1414), suggesting a lower degree of methylesterification in *frb1*. These results are congruent with the decreased PME transcript levels.

## Discussion

We identified a gene, *FRB1*, which encodes a Golgi-localized protein required for cell adhesion in *Arabidopsis*. Loss of FRB1 function leads to dramatic changes in cell dissociation and to ectopic cell adhesion. These opposing phenotypes imply that a fundamentally different mechanism is affected in *frb1* than in other previously reported cell adhesion-defective mutants. Although we have so far been unable to determine the molecular function of FRB1, below we consider a number of possibilities.

The alterations in cell adhesion could link FRB1 to either cuticle or cell wall metabolism. For example, loss of cuticle or wax at the cell surface could expose cell wall structures to each other, which could lead to cell fusions and separation of individual cells from the surface. The increase in penetration of toluidine blue (Figure S4) in leaf tissues of the *frb1–1* is consistent with loss of epidermal tissue integrity, and with damage to the cuticle that usually provides an effective hydrophobic barrier to stain penetration. However, it is difficult to imagine how this integrity-loss could lead to phenotypes such as complete organ breakage, where not just surface cells become separated but also cells in deeper tissues. Consistent with the latter, neither wax, nor cuticle defective mutants described so far have cell dissociation phenotypes [47]–[49]. In addition, most of the fatty acid processing leading up to wax production occurs in the endoplasmic reticulum [28] whereas FRB1 is located in the Golgi. Furthermore, several *frb1* mutant phenotypes are evident during embryo development where the influence of the cuticle is minimal. We therefore favor that the gene product affects cell wall components as discussed below, and that it is the gross tissue disruption that leads to cuticle cracking (Figure S6).

Although XyG (or more specifically the LM15 epitope XXXG [16]) co-aligns with cell wall micro-domains at the edge of the cell adhesion plane [17], there is no direct evidence that XyGs disrupt cell adhesion. Furthermore, none of the mutants affecting XyG biosynthesis (e.g. [50]–[54], [92]) or metabolism (e.g. [38], [55]–[57]) shows cell adhesion defects. Hence, while we observed changes in the XyG fine structure, we believe it is unlikely that the organ fusion or separation in *frb1* is directly associated with changes in XyG metabolism.

The decrease of galactose content in the Golgi, and consequently in the pectic cell wall fraction of *frb1*, suggests that a galactose-containing component is dependent on FRB1 function. Since it is improbable that a loss-of-function mutant would directly lead to an increase in arabinose incorporation, it is plausible that the remaining cell wall alterations are affected indirectly in *frb1*. Unbranched α-(1–5)-L-Ara and β-(1–4)-D-Gal, as well as the type I arabinogalactan side chains ([β-(1–4)-D-Gal]_n_ branched at O-3 with short α-(1–3)-L-Ara) of RGI, are turned over during fruit ripening and softening and are believed to play a role in natural cell adhesion and separation processes [58], although their specific roles are poorly understood. It is possible that the observed under-galactosylation in *frb1* may signify a loss in pectic galactan side chains, which could promote cell separation. In *Arabidopsis*, mutations in the RGI arabinosyltransferase, *ARAD1* [59] exhibit a decrease (∼50%) in pectic arabinose without showing any cell dissociation phenotype. Assuming that arabinan and galactan side chains fulfill related functions during cell adhesion we hypothesize that FRB1 is more likely involved in protein glycosylation than in RGI galactosylation. Glycosylation of proteins can occur at various subcellular sites [60]. The mechanism of O-glycosylation of AGPs is not well understood and FRB1 activity could be associated with AGP type II synthesis. In contrast to type I AGPs, the type II AGPs show more branched galactan structures (β-(1–3) or (1–6)-D-Gal), which can be terminated by α-(1–3)-L-Ara, α-(1–5)-L-Ara residues, or by less abundant sugars (reviewed in [61], [62]). Previously described mutations leading to cell adhesion deficiencies have been readily explained by reduced abundance of pectins [18], [19], [21], [23], [24], [63]. For example, in *qua1* mutants, reduced cell adhesion is accompanied by a 25% reduction in HG levels and reduced staining with HG specific antibodies [18], [63]. In contrast, we did not observe any reduction in uronic acid levels, and observed abundant pectic epitopes in *frb1*, suggesting that FRB1 function is not required for bulk pectin synthesis. However, a *PME* was up-regulated in the *frb1* mutants, which also corresponded with an increased PME activity in the mutants. FTIR analyses further revealed that the degree of methyl-esterification of pectins was changed, which can affect cell adhesion [19]. Changes in PME activity may result in both a decrease [19] and increase [64] in cell adhesive properties, depending on tissues and organs investigated.

In addition to the possibilities outlined above, FRB1 activity could influence a class of cell wall proteins that are required for cell adhesion. For example, several cell wall polymers contain both arabinose and galactose, including pectins (RGI) and cell wall glycoproteins. Immunolabeling revealed that the increased arabinose content appears to be mainly associated with extensins, i.e. as estimated using the LM1 antibody. While extensins have not been directly associated with cell adhesion, it was suggested that extensins cross-link and provide a template for pectic polymers [65], [66]. It could be that perturbations in extensin deposition and pectin methyl-esterification affect cell adhesion and fusion. Intriguingly, such scenario is supported by both *in vitro* and *in vivo* studies [65], [66]. *In vitro* measurements using atomic force microscopy and surface plasmon resonance techniques revealed that extensins and homogalacturonans can be intimately mixed, and that such interactions affect the surface of layered pectin polymers, and the water holding ability of the pectins [66]. Furthermore, the charge of pectin polymers, which may be related to the degree of esterification, and extensins can influence interactions between the two components. In such a scenario, certain polymers may become more accessible and perhaps even change their scaffolding to other wall polymers. This may thus explain why the surface of the *frb1* seedlings were coated with JIM5 and JIM7 decorated material, despite unaltered uronic acid levels in the wall. This implies that changes in the structure of the wall may cause the polymers to be less well integrated.

Finally, a possible function for FRB1 is suggested by remote homology searches with the FRB1 sequence using PSI-BLAST [69]. These searches retrieved sequences for protein O-fucosyltransferases (POFUT1) from a number of different plant species, including *Arabidopsis* (Figure S7). This type of fucosyltransferase is known to add fucose directly to Ser or Thr in glycoproteins [89]. However, demonstrating that the protein does indeed have this activity has so far eluded us. The primary limitation has been that we have not yet determined what the targets for this putative glycosyltransferase might be. In addition, although FRB1 has remote similarities to fucosyltransferases, we do not have any indication that this protein does indeed transfer fucose. Future work with FRB1 will therefore require systematic and exhaustive testing of both potential targets and substrates.

In summary, we propose that mutations in FRB1 lead to severe cell adhesion defects due to a previously unreported mechanism that affects aspect of cell wall and middle lamella architecture, including both the pectin and extensin matrices.

## Materials and Methods

### Plant material and growth conditions

All wild-type and mutant *Arabidopsis* were ecotype Columbia (Col-0). Seedlings were grown either under continuous light (200 µE/m^2^/s) at 21°C on plates containing half-strength Murashige and Skoog (MS) mineral salts (Sigma-Aldrich, St. Louis, MO) or on full strength MS medium complemented with 1% sucrose under a long day regime (16 h light [200 µE/m^2^/s], 8 h darkness). Both were solidified with 0.8% agar.

### Cloning of *FRB1*

T-DNA insertion in *frb1–1* plants was mapped by thermal asymmetric interlaced (TAIL-) PCR according to [67]. The insertion-specific PCR product was sequenced (700 bp upstream of the At5g01100 coding region), and confirmed by PCR (Table S3). Additional insertions at the At5g01100 locus SALK_078459 (*frb1–2*) and WiscDsLox1D5 (*frb1–3*), were obtained from the Arabidopsis Biological Resource Center. The sites of insertion were confirmed by PCR (Table S3). Transcript levels of *FRB1* were assessed by RT-PCR (Table S3).

### Bioinformatic analysis

FRB1 homologies were identified by BLAST [68], [69]; retaining hits with E-values less than 10^−20^. MEGA4.0 (Molecular Evolutionary Genetics Analysis) was used for both sequence alignment and phylogeny tree building [70], which were further compared by a neighbor-joining method [71]. JTT matrix-based method [72] was used to compute evolutionary distance.

### Microscopy

Whole seedling micrographs were captured on a Leica MZ16 stereomicroscope equipped with a DFC320 camera. Confocal images were acquired using a Nikon inverted microscope equipped with a BioRad MRC 1024 confocal head with a krypton-argon laser. All images were compiled and analyzed using the BioRad software package LaserSharp (BioRad, Hercules, CA) and NIH Image (Wayne Rasband, RSB, NIH, Bethesda Maryland). Z-series were obtained by collecting 20–25 µm deep series (typically 1 µm steps). Movies with GFP::FRB1 transgenic lines were created by collecting images every 3 seconds for a total of 40 images and converted to time lapse movies (10 frames per second). Embryos were stained with analine blue and imaged as previously described [73].

A method for RNA *in situ* hybridization previously described by Weigel and Glazebrook [74] was modified for embedding of hypocotyls, subsequent transversal sectioning and LM1 and LM6 immunolabeling. Ten µm tissue sections were prepared in paraffin, washed and rehydrated in an ethanol series. Non-specific antibody binding was blocked by incubation in blocking solution (phosphate-buffered saline (PBS) +3% (w:w) fat free milk powder). LM1 and LM6 antibodies (Plant Probes, UK) were diluted 1∶5 in blocking solution and incubated overnight at 4°C. Excess of antibodies were washed three times with PBS for 15 min each. Sections were incubated with goat anti-rat conjugated to Alexafluor-488 (Molecular Probes, USA) diluted 2000-fold in 3% skim milk in PBS for 1 h in the dark. Samples were washed three times with PBS buffer for15 min each prior to visualization.

For whole mount immuno-localization, unfixed 5 day old seedlings were incubated in JIM5 or JIM7 rat monoclonal antibody (CarboSource, USA) diluted 10-fold in 5% skim milk in PBS for 1 hour; followed by 5 washes in excess PBS and incubation for 1 hour in goat anti-rat secondary antibody conjugated to Alexafluor-488 (Molecular Probes, USA), diluted 500-fold in 5% skim milk in PBS. After washing three times in PBS, seedlings were mounted in AF3 antifade (Citifluor, UK) and viewed with a confocal microscope. Seedling tissues were prepared for scanning microscopy and sections prepared for immuno-localization as previously described [75].

### Cell wall extraction and determination of composition

Neutral sugars, uronic acids and cellulose were determined in 10-day-old seedlings grown on MS-media in long day regime. Plant material was flash-frozen, ground and washed with 70% ethanol (thoroughly vortexed, spun down 14.000 rpm, 10 min Eppendorf table centrifuge). The pellet was washed with chloroform∶methanol (1∶1 v/v) as above and was air dried (CWM). Cell wall polymers and cell wall precursors (oligosaccharides) were acid hydrolyzed prior to sugar analysis. Fractionation was performed as described in [76]. In detail, plants were harvested at the end of the night to avoid starch contamination and cell wall material was obtained as described above. Cell wall material (10 mg) was resuspended in 1.5 ml CDTA (50 mM), vortexed and mixed for 6 h at 1100 rpm and RT on an Eppendorf thermo shaker. The samples were precipitated (14000 rpm, 10 min, Eppendorf table centrifuge) and the supernatants were stored at −20°C. The pellet was resolved in 1.5 ml CDTA again and incubated overnight. Samples were centrifuged and the supernatant was combined with the other CDTA fraction and stored at −20°C. The procedure was repeated by resolving and incubating the pellet in 1.5 ml Na_2_CO_3_ (50 mM) as described above. The corresponding Na_2_CO_3_ fractions were combined and stored at −20°C. A third round of extraction was applied by using 1.5 ml of KOH (4 M) as described for the CDTA and Na_2_CO_3_ fractions. After combining the two KOH fractions and their storage at −20°C the pellet was washed three times with water and subsequent centrifugation (10 min, 14000 rpm) to remove excess of KOH. The fractions were dialyzed (Spectra/Por3 Dialysis Membrane, MWCO 3500, Spectrum Laboratories) for two days against three exchanges of 3 L of water at 4°C and under constant shaking before they were flash frozen and freeze dried. Matrix polymers were hydrolyzed by 2 M TFA (1 h, 121°C) and neutral sugars measured as described in [38] modified from [77]. Uronic acids were measured by the m-hydroxybiphenyl/H_2_SO_4_ method [78]. To remove 4 M KOH insoluble matrix polysaccharides the pellet was treated with 2 M TFA and the remnant was considered as cellulose. Cellulose was measured as the amount of hexoses released by sulphuric acid under constant stirring (72%, 1 h, RT) treatment. Hexoses were determined by anthrone assay [79].

Sugar data derived from microsomal preparations were obtained by a modified version from [38] using a Dionex HPAEC system (ICS 3000) equipped with a CarboPac PA20 column and PAD detection system (Dionex, Sunnyvale, CA, USA). The following gradient was applied comprising three phases of isocratic flow of aqueous NaOH solution (0–20 min: 8 mM, 20–46 min: 771 mM, 46–62 min: 8 mM; flow rate 0.40 ml/min).

For the sequential extraction of uronic acids, 10-day-old seedlings (as above) were treated with 70% ethanol for 1 h at 70°C. Whole seedlings were then transferred to chloroform∶methanol (1∶1 v/v; 4 h), followed by acetone wash. Seedlings were air dried for 48 h before dry weight measurements were performed. Pectin was sequentially extracted (5–10 mg of dry tissue) with 0.5% ammonium oxalate (25°C, 2 h), washed with water (2 X), and extracted with 1 M H_2_SO_4_ (100°C, 1 h). Uronic acids were measured as mentioned above [78]. Determination of the XyG fine structure by Oligosaccharide Mass Profiling (OLIMP) was performed as described earlier by [38] and references within [80], [81].

### GUS expression analysis

A genomic DNA fragment extending ∼4.3 kb upstream of the ATG starting codon for *FRB1* was cloned in front of the GUS gene in pCAMBIA1305.1 ([82]; Table S3), transformed into plants by *Agrobacterium*-mediated transformation [83], and GUS analyses were performed [76] on T3 generation. Tissues were observed for GUS staining under a dissecting microscope.

### Generation of EGFP-FRB1

A full length cDNA of *FRB1* was obtained from the Riken Bioresource Center (Japan), and was sub-cloned into pZERO (Invitrogen; Table S3). The *FRB1* cDNA was excized using EcoRV and BamHI and the sub-cloned into the SmaI and BamHI sites of the pEGAD [84] to create an in frame fusion of EGFP with FRB1. This construct was transformed into Arabidopsis plants by the floral dip method [85] and selected on soil using 0.25 g/L Basta. T1 plants were screened for EGFP expression, and etiolated T2 seedlings were visualized by confocal microscopy. Transient co-expression of EGFP-FRB1 and m-Cherry Golgi marker (CD3–967) [33] in N. benthamiana was performed according to [19]. Colocalization EGFP-FRB1 and CD3–967 was captured by a single laser pass at 100 Hz using a Leica TCS SP5 confocal microscope using excitation for EGFP and mCherry at 488 nm and 584 nm respectively.

### Microarray hybridizations and analysis

Three replicates of FRB1 and *frb1–1* RNA were prepared from 10-day-old seedlings using Trizol reagent (Invitrogen) according to the manufacturer's recommendations. RNA processing and cRNA hybridizations to the Affymetrix ATH1 gene chip were performed according to a modified Affymetrix protocol developed at the University of Toronto, Department of Cell and Systems Biology Affymetrix Genechip Facility (http://www.csb.utoronto.ca/resources/). All data is MIAME compliant and has been deposited in the Gene Expression Omnibus (GEO) database. The accession number for the full dataset is GSE31033.

Raw hybridization data was normalized using MAS 5.0 with a TGT of 500. Any absent probesets from any of the replicates were removed from the dataset. Further analysis was done using MS Excel. AtGenExpress stress datasets [86] (heat, drought, osmotic, oxidative) were downloaded from the NASCArrays website (http://affymetrix.arabidopsis.info/). For the four stress datasets, the up-regulated genes were extracted from each of the time point (0.5, 1, 3, 6, 12 and 24-hour treatment) separated. The final list of *frb1–1* up-regulated list was compared with that of each of the four stress datasets, and the genes that are up-regulated in both were identified.

### FTIR measurements

Ground cell wall material of 10 day old light grown seedlings (n≥5) was used to measure its transmittance by a Varian 1000 FTIR. Each FTIR spectrum was recorded from 1800 to 800 cm^−1^. Curves were normalized by subtraction of the minimum value from each data point and the areas below the graph were transformed into relative arbitrary units by dividing each data point by the maximum value. Absorbance was calculated by inverting transmittance to its reciprocal. Five wild-type samples (n = 5) were averaged whereas minimum two independent samples of *frb1–1*, *frb1–2* and *frb1–3* each were used to express the average relative absorbance (n = 7).

### Cuticle integrity analysis

*Arabidopsis thaliana* ecotype Columbia (Col-0) and the *frb1–1* mutant were grown on half-strength MS salts, solidified with 0.8% agar, under continuous light. 28 days after vernalization plants were gently rinsed with water to remove agar, then were subjected to the toluidine blue staining test as described by [87].

### PME activity assays

Pectin methylesterase (PME) activity was measured using an alcohol oxidase coupled assay on 5-day-old light-grown (24 h) seedlings according to [88]. The PME activity is calculated as the activity releasing one mole of methanol per second (1 kat).

### FRB1 activity assays

FRB1 over-expression in insect cells we carried out using the BaculoDirect baculovirus expression system from Invitrogen Corp. A full length *FRB1* entry clone was PCR generated using *FRB1* forward (5′-CACCATGTCAGTCGGCGTTCCAGTG) and reverse (5′-TTATCTCAGAGATTGTGCTCGTA) primers designed for use with the pENTR//D- TOPO cloning kit from Invitrogen. The entry clone was then used to create a baculovirus expression clone with an N-terminal histidine/V5 epitope tag. Sf9 cells were maintained and infections were carried out according to the manufacturer's recommendations. Typically, cells were harvested 3 days post-infection from 75 cm^2^ cell culture dishes by centrifugation and protein was extracted by incubating the cells in 0.5 mL cold lysis buffer (50 mM Hepes, pH 7.3, 1% TritonX-100, 150 mM NaCl, 1 mM EDTA, 1 mM PMSF) for 30 minutes with shaking. Protein concentrations were determined using a bicinchoninic acid (BCA) protein assay (Thermo Scientific Pierce). Standard assays contained 100 µg protein extract, 50 mM Hepes pH 7.6, 0.2 M sucrose, 0.05% BSA, 100 µM UDP/GDP-sugar, 100 µg acceptor (see below), 1 µM [^3^H]UDP/GDP-sugar (specific activity 60 Ci/mmol),1 mM MnCl_2_ in a final volume of 50 µL. Samples were incubated for 1 h at 30°C. Reactions were terminated by adding 900 µL of chloroform∶methanol (3∶2 v/v) and centrifuged at 10 000 *g* for 5 min. The pellet was washed by re-suspending in 70% ethanol, sonicating for 15 min and centrifuging at 10 000 *g*. This process was repeated at least three more times. The final pellet was resuspended in 200 µL of 10 mM EDTA and counted by liquid scintillation. Values in Table S1 were calculated based on total counts per minute for FRB1 expressing cells and non-infected controls. In all cases, the acceptor was a membrane protein preparation made from 5 day-old *FRB1* seedlings. Membranes were prepared according to a modified protocol from[90]. Briefly, plant tissue was homogenized using a mortar and pestle in griding buffer (250 mM sucrose, 50 mM Hepes, pH 7.5, 25 mM KCl, 5 mM EDTA , 1 mM PMSF) at 4C. The homogenate was centrifuged at 1000 *g* for 10 min and the supernatant was retained. The supernatant was centrifuged at 10 000 *g* for 10 min and the supernatant was retained. This supernatant was then centrifuged at 100 000 *g* for 60 min at 4C. Following centrifugation the supernatant was transferred to a fresh tube (soluble fraction) and the pellet resuspended in 50 mM Hepes, pH 7.5 using a glass homogenizer and centrifuged again at 100 000 *g* for 60 min to yield a pellet (membrane fraction) that was used for subsequent assays. When this membrane preparation was used as acceptor it was heat inactivated at 60°C for 20 min prior to addition to reactions. For the sugar incorporation assays the membrane preparation was used immediately. For these assays, membranes were resuspended in buffer containing 50 mM Hepes pH 7.6, 100 mM KCl, 5 mM MgCl2, 5 mM MnCl2, 100 µg of protein, 1 µM [H^3^]UDP-sugar (specific activity 60 Ci/mmol). Triplicates of samples were incubated at 30°C for 1 hour. The samples were extracted using chloroform∶methanol (2∶1) followed by 4 times sequential addition of equal volumes of water. The organic phase was collected and proteins precipitated by adding acetone and washing the pellet is acetone three times. The organic phase represents the “lipid” fraction. Total radioactivity was measured by liquid scintillation.

### Western blotting

Protein from tobacco leaves expressing GFP-FRB1 or CD3-967 were fractionated into soluble and membrane fractions according to the protocol described above. Western blotting was done using standard techniques [91] using anti-GFP (Bioshop, Canada) and anti-mCherry (Biovisions).

## Supporting Information

Figure S1 **Locations of T-DNA insertions in *frb1* alleles, *FRB1* expression and FRB1 predicted protein structure. **A. Relative positions of T-DNA insertions in *frb1-1*, *frb-1-2* and *frb1-3* lines. B. RT-PCR using either FRB1 specific primers (FRB1) or actin specific primers (ACT7) with *FRB1*, *frb1-1*, *frb1-2*, or *frb1-3* cDNA as template. Number of PCR cycles are indicated. C. Predicted transmembrane and DUF246 domain positions in FRB1 protein.
(TIF)

Figure S2 **Phylogenetic relationships of FRB1 and its homologues in the *Arabidopsis* genome. **Evolutionary history was inferred using the Neighbor-Joining method. The optimal tree with the sum of branch length = 14.99 is shown. The percentage of replicate trees in which the associated taxa clustered together in the bootstrap test (1000 replicates) is shown next to the branches. The tree is drawn to scale, with branch lengths in the same units as those of the evolutionary distances used to infer the phylogenetic tree. The evolutionary distances were computed using the JTT matrix-based method and are in the units of the number of amino acid substitutions per site. All positions containing alignment gaps and missing data were eliminated only in pairwise sequence comparisons.
(TIF)

Figure S3 **Expression of the *FRB1* gene. A. to I. **The *FRB1* **expression was examined using the 4.3 kbp promoter sequence just upstream of the** *FRB1* **start codon fused to GUS. A. to E. GUS activity in one- (A.), two- (B.), three- (C.), four- (D.), and five-day-old (E.) light grown seedlings, respectively. F. to I. GUS activity in two- (F.), three- (G.), four- (H.), and five-day-old (I.) etiolated seedlings, respectively.**
(TIF)

Figure S4 **Functional complementation of** ***frb1*** **mutant phenotype by over-expression of GFP-FRB1 fusion protein in seedlings.** A. *FRB1* seedling. B. *GFP-FRB1*; *frb1-2* seedling. C. *frb1-2* seedling. D. *frb1-2* seedling.
(TIF)

Figure S5 **Western blot of GFP-FRB1 and mCherry Golgi marker.** Blot of proteins from tobacco cells expressing either GFP-FRB1 or CD3-967. Lanes 1 and 2 are probed with anti-GFP antibody and lanes 3 and 4 are probed with anti-mCherry antibody. Lanes 1 and 3 are soluble proteins and lanes 2 and 4 are membrane proteins. The faint low molecular weight band in lane 2 is likely a cleavage product of the GFP-FRB1 fusion.
(TIF)

Figure S6 **Penetration of toluidine blue into cotyledons. **A. *FRB1* seedling. B. *frb1* seedling. Scale bars: 2 mm.****
(TIF)

Figure S7 **Alignment of FRB1 to protein O-fucosyltransferases (POFUT1) from different plant species.** **Black boxes are identical and grey boxes are similar amino acids.**
(TIF)

Movie S1 **Time lapse movie of GFP-FRB1 particles in dark grown hypocotyl cells over 2 minutes.** Scale bar equals 10 µm.
(MOV)

Movie S2 **Time lapse movie of GFP-FRB1 particles in dark grown hypocotyl cells over 2 minutes following treatment with 1 µM Latrunculin B for 15 min.** Scale bar equals 10 µm.
(MOV)

Movie S3 **Time lapse movie of GFP-FRB1 particles in dark grown hypocotyl cells over 2 minutes following treatment with 20 µM oryzalin for 30 min.** Scale bar equals 10 µm.
(MOV)

Table S1 **Percent incorporation of activated sugars by FRB1 expressing insect cell extracts compared to extracts of uninfected controls. **Values represent the average percent incorporation for at least six replicate reactions containing FRB1 protein.
(DOC)

Table S2 **Genes with altered expression in a** ***frb1-1*** **background.**
(XLSX)

Table S3 **List of primers used in this study.**
(DOC)

Table S4 ***frb1*** **-upregulated genes that are also upregulated by stress treatments.** Abbreviations: S, shoot; R, root.
(DOC)
