# Supplementary figures and images for: The FRIABLE1 Gene Product Affects Cell Adhesion in Arabidopsis
Source: PLoS One. 2012 Aug 14;7(8):e42914. doi: 10.1371/journal.pone.0042914 (PMC3419242; doi:10.1371/journal.pone.0042914)

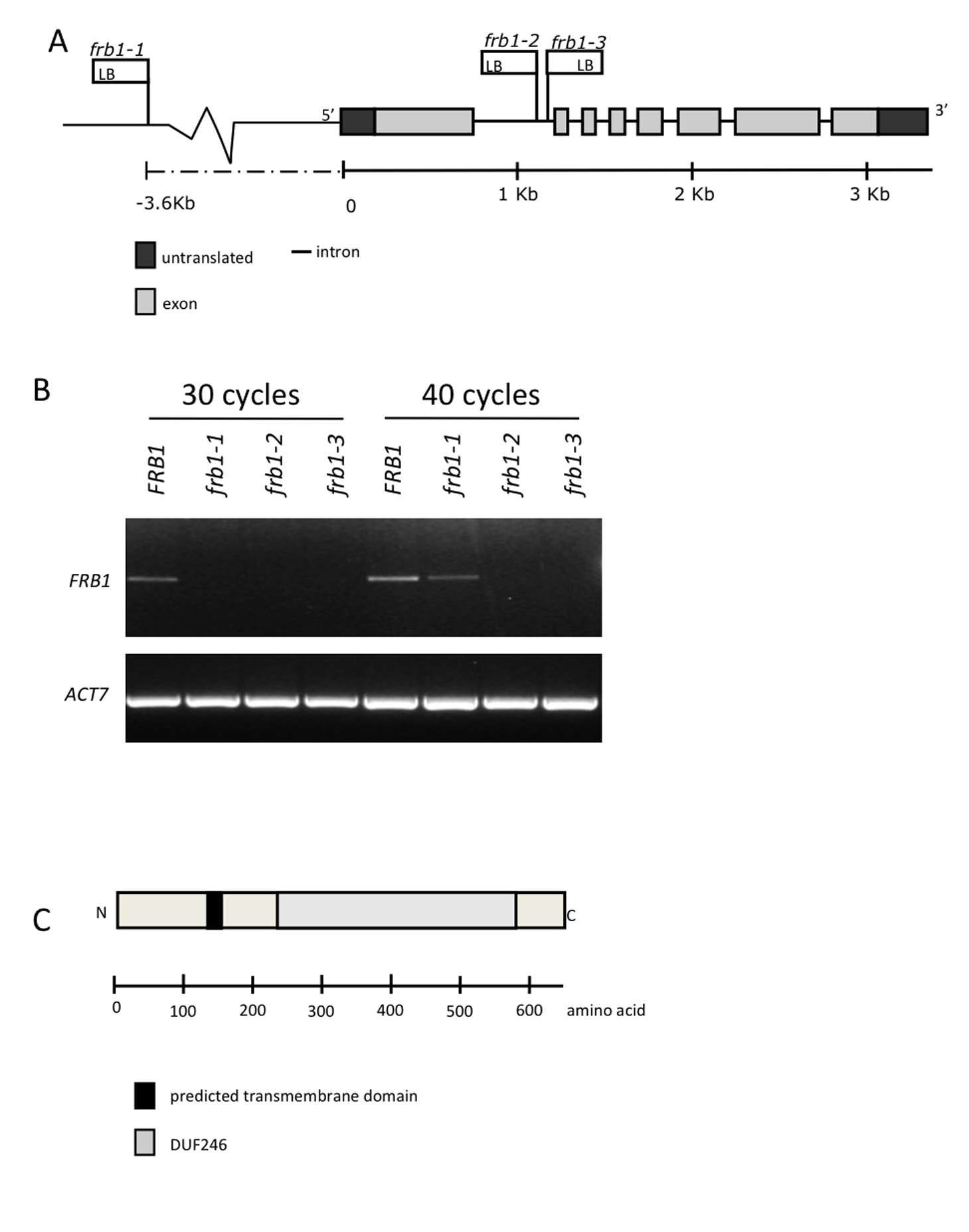

Supplement: Figure S1 — Locations of T-DNA insertions in frb1 alleles, FRB1 expression and FRB1 predicted protein structure. A. Relative positions of T-DNA insertions in frb1-1, frb-1-2 and frb1-3 lines. B. RT-PCR using either FRB1 specific primers (FRB1) or actin specific primers (ACT7) with FRB1, frb1-1, frb1-2, or frb1-3 cDNA as template. Number of PCR cycles are indicated. C. Predicted transmembrane and DUF246 domain positions in FRB1 protein. (TIF) [file pone.0042914.s001.tif]

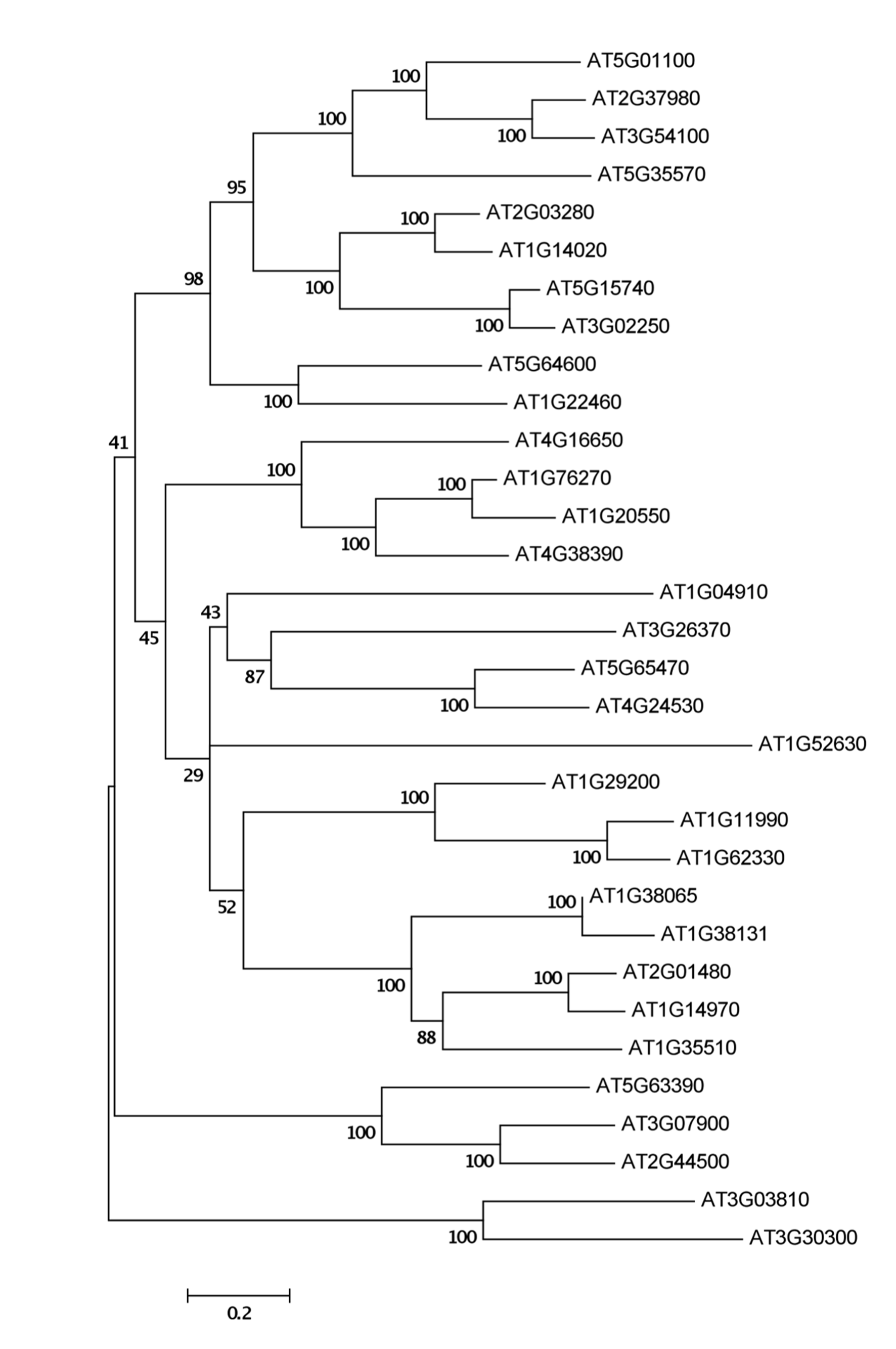

Supplement: Figure S2 — Phylogenetic relationships of FRB1 and its homologues in the Arabidopsis genome. Evolutionary history was inferred using the Neighbor-Joining method. The optimal tree with the sum of branch length = 14.99 is shown. The percentage of replicate trees in which the associated taxa clustered together in the bootstrap test (1000 replicates) is shown next to the branches. The tree is drawn to scale, with branch lengths in the same units as those of the evolutionary distances used to infer the phylogenetic tree. The evolutionary distances were computed using the JTT matrix-based method and are in the units of the number of amino acid substitutions per site. All positions containing alignment gaps and missing data were eliminated only in pairwise sequence comparisons. (TIF) [file pone.0042914.s002.tif]

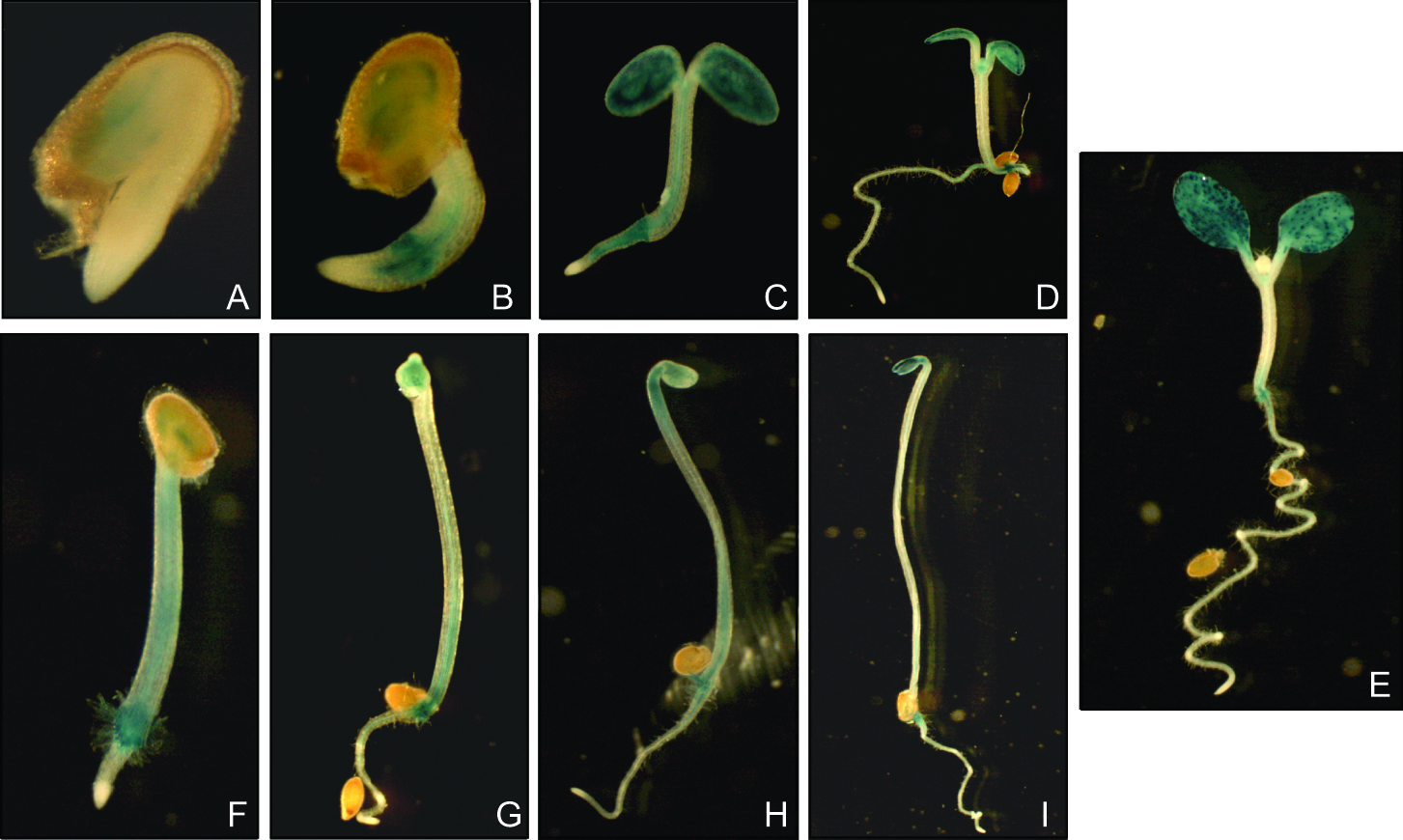

Supplement: Figure S3 — Expression of the FRB1 gene. A. to I. The FRB1 expression was examined using the 4.3 kbp promoter sequence just upstream of the FRB1 start codon fused to GUS. A. to E. GUS activity in one- (A.), two- (B.), three- (C.), four- (D.), and five-day-old (E.) light grown seedlings, respectively. F. to I. GUS activity in two- (F.), three- (G.), four- (H.), and five-day-old (I.) etiolated seedlings, respectively. (TIF) [file pone.0042914.s003.tif]

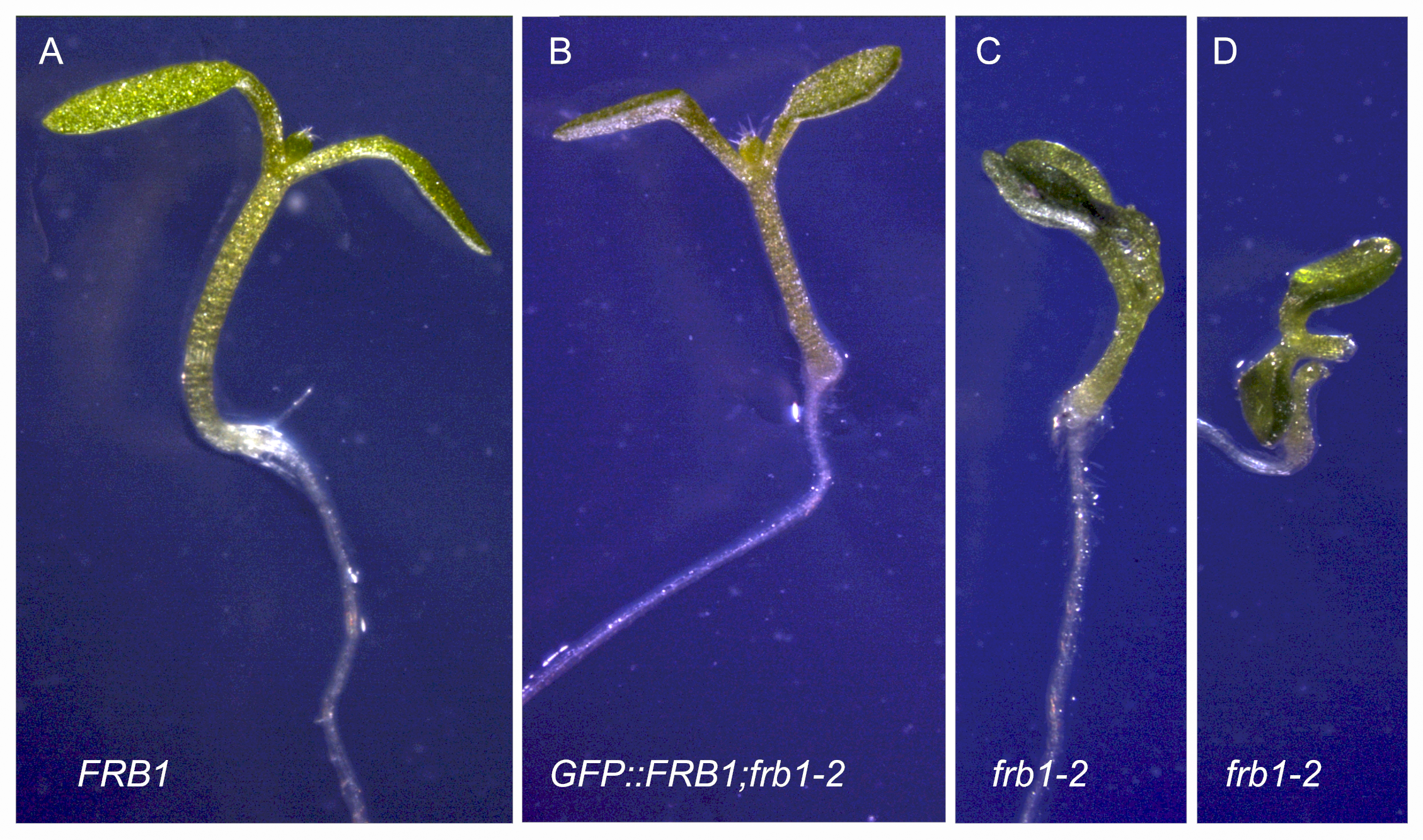

Supplement: Figure S4 — Functional complementation of frb1 mutant phenotype by over-expression of GFP-FRB1 fusion protein in seedlings. A. FRB1 seedling. B. GFP-FRB1; frb1-2 seedling. C. frb1-2 seedling. D. frb1-2 seedling. (TIF) [file pone.0042914.s004.tif]

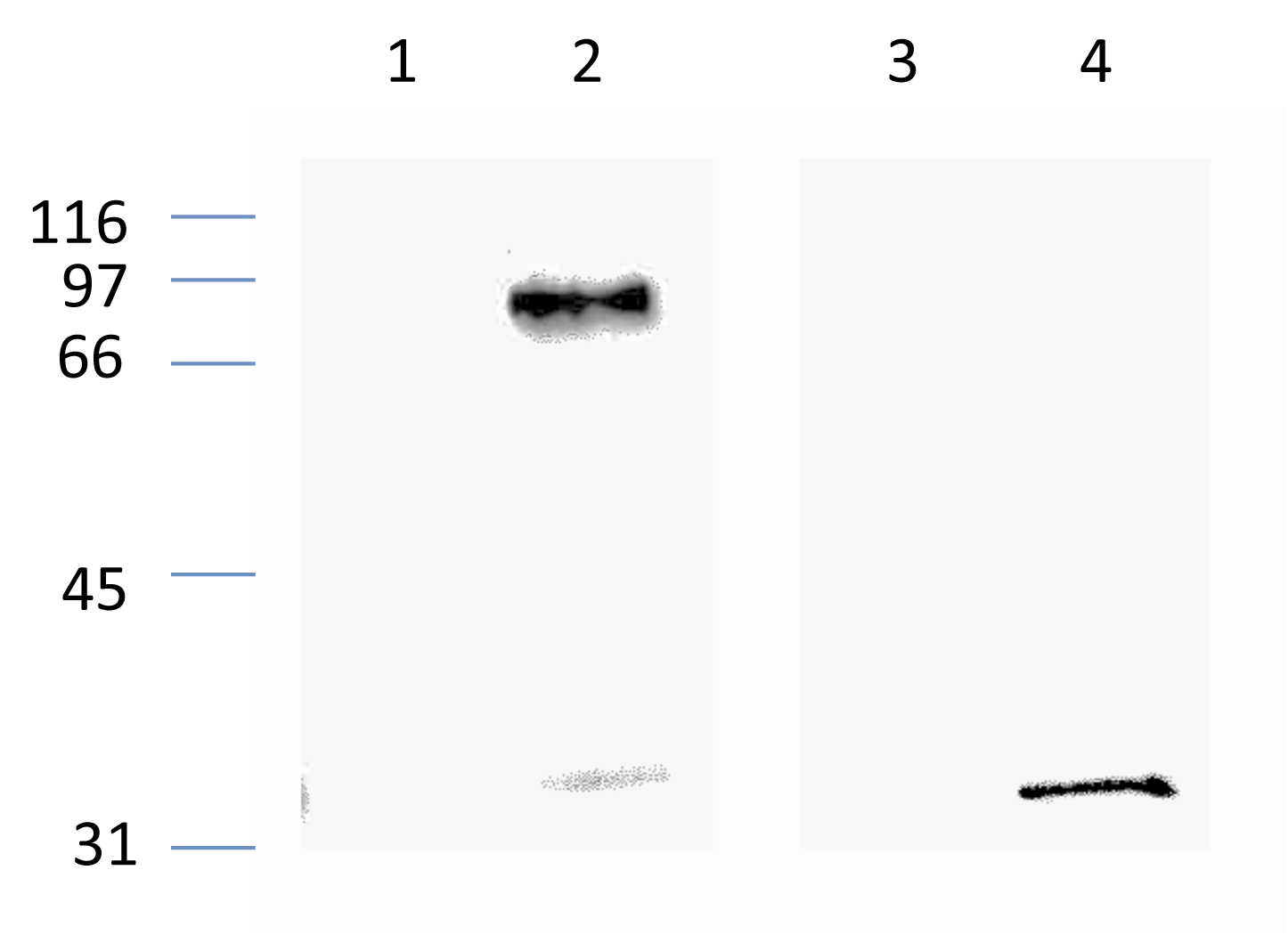

Supplement: Figure S5 — Western blot of GFP-FRB1 and mCherry Golgi marker. Blot of proteins from tobacco cells expressing either GFP-FRB1 or CD3-967. Lanes 1 and 2 are probed with anti-GFP antibody and lanes 3 and 4 are probed with anti-mCherry antibody. Lanes 1 and 3 are soluble proteins and lanes 2 and 4 are membrane proteins. The faint low molecular weight band in lane 2 is likely a cleavage product of the GFP-FRB1 fusion. (TIF) [file pone.0042914.s005.tif]

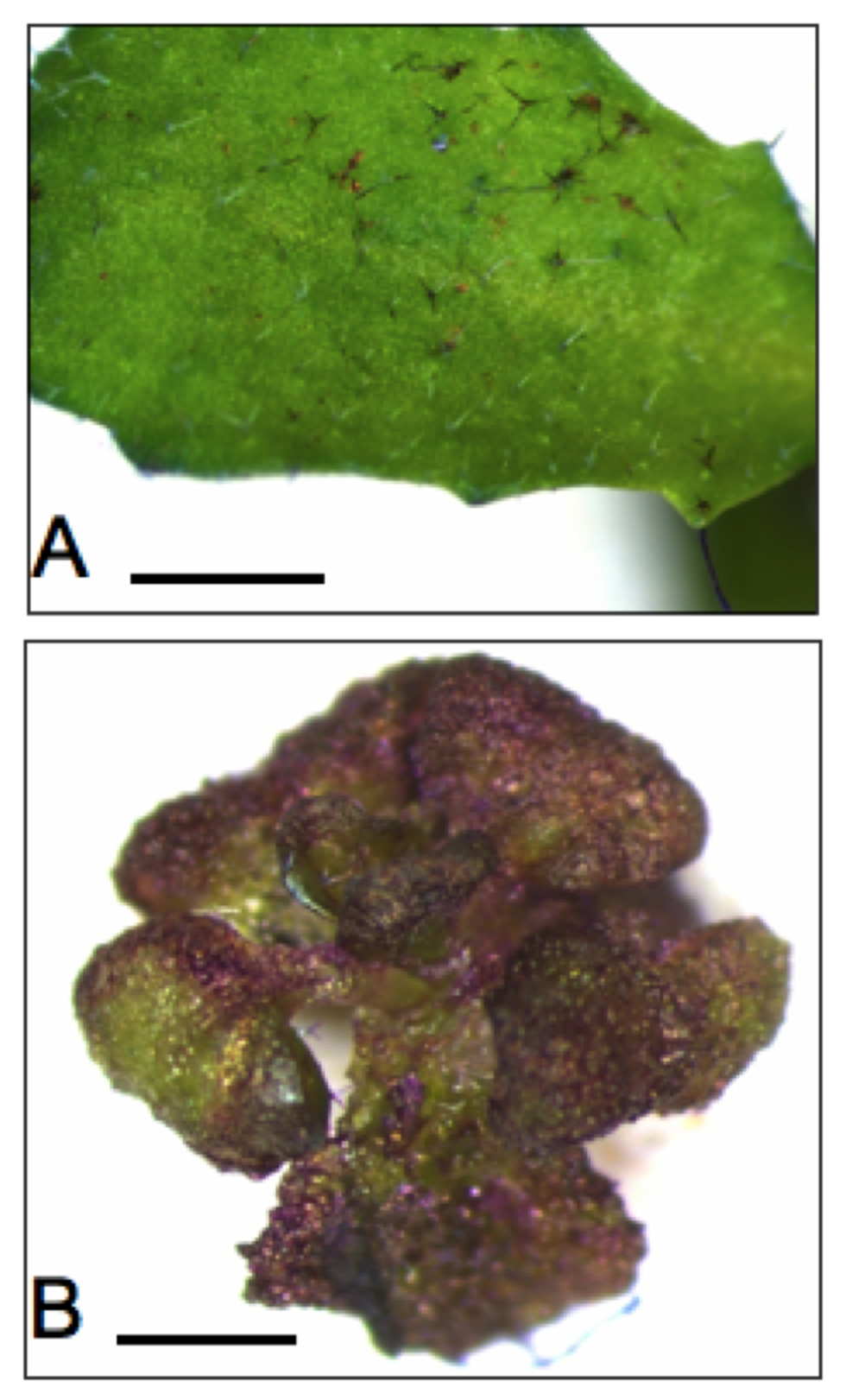

Supplement: Figure S6 — Penetration of toluidine blue into cotyledons. A. FRB1 seedling. B. frb1 seedling. Scale bars: 2 mm. (TIF) [file pone.0042914.s006.tif]

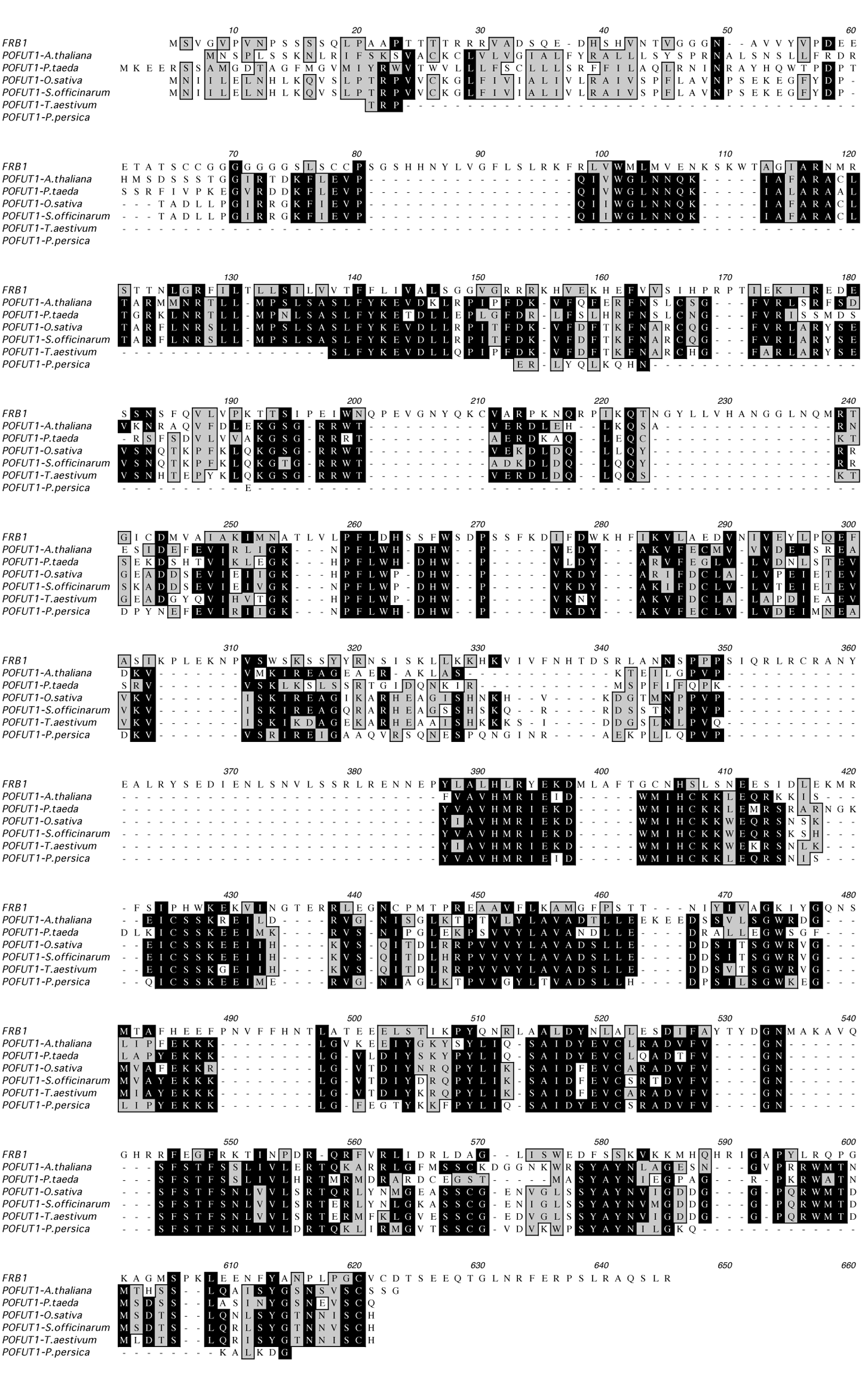

Supplement: Figure S7 — Alignment of FRB1 to protein O-fucosyltransferases (POFUT1) from different plant species. Black boxes are identical and grey boxes are similar amino acids. (TIF) [file pone.0042914.s007.tif]
