# Supplementary material for: The FRIABLE1 Gene Product Affects Cell Adhesion in Arabidopsis
Source: PLoS One. 2012 Aug 14;7(8):e42914. doi: 10.1371/journal.pone.0042914 (PMC3419242; doi:10.1371/journal.pone.0042914)
Supplement: Table S1 — Percent incorporation of activated sugars by FRB1 expressing insect cell extracts compared to extracts of uninfected controls. Values represent the average percent incorporation for at least six replicate reactions containing FRB1 protein. (DOC) [file pone.0042914.s011.doc]

Table S1.

| Activated sugar | Percent incorporation compared to control | Standard deviation (+/-) |
| --- | --- | --- |
| UDP-Glc | 102.5 | 18.1 |
| UDP-Gal | 69.3 | 13.8 |
| GDP-Man | 72.7 | 18.9 |
| GDP-Fuc | 62.8 | 11.1 |
| UDP-GlcNAc | 88.4 | 10.4 |
| UDP-GalA | 61.1 | 24.8 |
| UDP-GlcA | 61.8 | 11.1 |
