# Supplementary material for: The FRIABLE1 Gene Product Affects Cell Adhesion in Arabidopsis
Source: PLoS One. 2012 Aug 14;7(8):e42914. doi: 10.1371/journal.pone.0042914 (PMC3419242; doi:10.1371/journal.pone.0042914)
Supplement: Table S3 — List of primers used in this study. (DOC) [file pone.0042914.s013.doc]

Table S3.

| Primer | Sequence |
| --- | --- |
| Primers used for RT-PCR. | |
| FRB1-F | 5’ CTTCCGTTTCTTGACCATTCATC |
| FRB1-R | 5’ CTCAGAGATTGTGCTCGTAGACTTGG |
| ACT7-F | 5’ GGTGAGGATATTCAGCCACTTGTCTG |
| ACT7-R | 5’ TGTGAGATCCCGACCCGCAAGATC |
|  |  |
| Primers used to generate FRB1-promoter GUS fusions. | |
| GUSBAMH-F | 5' GCGGATCCTGAATCAGTATATTTTGGATTCTT |
| GUSNCO-R | 5' CATGCCATGGTGACCGCCGGGGAAAAC |
|  |  |
| Primers used to generate GFP::FRB1 translational fusions. | |
| FRBECORVF | 5' CTCGAGGATATCATGTCAGTCGGCGTTCCAGTGAATCCGTCAAGCAG |
| FRBBAMHR | 5' TCTAGAGGATCCTTATCTCAGAGATTGTGCTCGTAGACTTGGTCTTTC |
|  |  |
| Primers used to generate FRB1 baculovirus expression construct. | |
| BACFRB1-F | 5' CACCATGTCAGTCGGCGTTCCAGTG |
| BACFRB1-R | 5' TTATCTCAGAGATTGTGCTCGTA |
