# Supplementary material for: The FRIABLE1 Gene Product Affects Cell Adhesion in Arabidopsis
Source: PLoS One. 2012 Aug 14;7(8):e42914. doi: 10.1371/journal.pone.0042914 (PMC3419242; doi:10.1371/journal.pone.0042914)
Supplement: Table S4 — frb1 -upregulated genes that are also upregulated by stress treatments. Abbreviations: S, shoot; R, root. (DOC) [file pone.0042914.s014.doc]

Table S4.

| Probe Set | AGI | Modified TAIR 7.0 Annotation | Drought | Heat | Osmotic |
| --- | --- | --- | --- | --- | --- |
| 261175_at | At1g04800 | glycine-rich protein |  |  |  |
| 258939_at | At3g10020 | similar to Os12g0147200 [Oryza sativa (japonica cultivar-group)] (GB:NP_001066153.1); similar to Os11g0149200 [Oryza sativa (japonica cultivar-group)] (GB:NP_001065754.1) |  | R | S, R |
| 261526_at | At1g14370 | APK2A__APK2A (PROTEIN KINASE 2A); kinase |  |  |  |
| 266165_at | At2g28190 | CSD2_CZSOD2__CSD2 (COPPER/ZINC SUPEROXIDE DISMUTASE 2); copper, zinc superoxide dismutase |  |  |  |
| 255753_at | At1g18570 | MYB51_BW51A_BW51B__MYB51 (myb domain protein 51); DNA binding / transcription factor |  |  |  |
| 247488_at | At5g61820 | similar to hypothetical protein [Trifolium pratense] (GB:BAE71234.1); contains InterPro domain Stress up-regulated Nod 19; (InterPro:IPR011692) | S | R | S, R |
| 245692_at | At5g04150 | basic helix-loop-helix (bHLH) family protein |  |  |  |
| 245734_at | At1g73480 | hydrolase, alpha/beta fold family protein | S | R | S, R |
| 262137_at | At1g77920 | bZIP family transcription factor |  |  |  |
| 255926_at | At1g22190 | AP2 domain-containing transcription factor, putative | S | R | S, R |
| 256522_at | At1g66160 | U-box domain-containing protein | R |  |  |
| 263809_at | At2g04570 | GDSL-motif lipase/hydrolase family protein |  |  |  |
| 259751_at | At1g71030 | ATMYBL2__ATMYBL2 (Arabidopsis myb-like 2); DNA binding / transcription factor |  |  |  |
| 248870_at | At5g46710 | zinc-binding family protein |  | R |  |
| 261564_at | At1g01720 | ANAC002_ATAF1__ATAF1 (Arabidopsis NAC domain containing protein 2); transcription factor | S | R | S, R |
| 246595_at | At5g14780 | FDH__FDH (FORMATE DEHYDROGENASE); NAD binding / cofactor binding / oxidoreductase, acting on the CH-OH group of donors, NAD or NADP as acceptor |  |  | S, R |
| 257832_at | At3g26740 | CCL__CCL (CCR-LIKE) |  |  | R |
| 250435_at | At5g10380 | zinc finger (C3HC4-type RING finger) family protein | R |  | S |
| 265817_at | At2g18050 | HIS1-3__HIS1-3 (HISTONE H1-3); DNA binding |  |  |  |
| 246238_at | At4g36670 | mannitol transporter, putative | S, R |  |  |
| 249775_at | At5g24160 | squalene monooxygenase 1,2 / squalene epoxidase 1,2 (SQP1,2) |  |  |  |
| 266462_at | At2g47770 | benzodiazepine receptor-related |  |  |  |
| 245638_s_at | At1g24822 At1g24996 At1g25097 At1g25170 | similar to unknown protein [Arabidopsis thaliana] (TAIR:AT1G25097.1); similar to unknown protein [Arabidopsis thaliana] (TAIR:AT1G25170.1) |  |  |  |
| 251221_at | At3g62550 | universal stress protein (USP) family protein |  |  |  |
| 251705_at | At3g56400 | WRKY70__WRKY70 (WRKY DNA-binding protein 70); transcription factor | S |  | S |
| 264741_at | At1g62290 | pepsin A |  |  |  |
| 254042_at | At4g25810 | XTR6__XTR6 (XYLOGLUCAN ENDOTRANSGLYCOSYLASE 6); hydrolase, acting on glycosyl bonds |  |  |  |
| 265053_at | At1g52000 | jacalin lectin family protein |  |  |  |
| 245757_at | At1g35140 | PHI-1__PHI-1 (PHOSPHATE-INDUCED 1) |  |  |  |
| 257890_s_at | At3g17070 At3g42570 | peroxidase, putative |  |  |  |
| 263443_at | At2g28630 | beta-ketoacyl-CoA synthase family protein |  |  | S |
| 249101_at | At5g43580 | serine-type endopeptidase inhibitor |  |  |  |
| 251745_at | At3g55980 | zinc finger (CCCH-type) family protein | S, R |  | S |
| 260856_at | At1g21910 | AP2 domain-containing transcription factor family protein |  |  |  |
| 263216_s_at | At1g30720 At1g30730 | FAD-binding domain-containing protein |  |  |  |
| 261193_at | At1g32920 | similar to unknown protein [Arabidopsis thaliana] (TAIR:AT1G32928.1) | R |  | S |
| 252570_at | At3g45300 | IVD__IVD (ISOVALERYL-COA-DEHYDROGENASE) | S | S, R | S, R |
| 256464_at | At1g32560 | late embryogenesis abundant group 1 domain-containing protein / LEA group 1 domain-containing protein |  |  |  |
| 258063_at | At3g14620 | CYP72A8__CYP72A8 (cytochrome P450, family 72, subfamily A, polypeptide 8); oxygen binding |  | R | S, R |
| 246187_at | 246187_at |  |  |  |  |
| 250868_at | At5g03860 | malate synthase, putative |  |  |  |
| 255733_at | At1g25400 | similar to unknown protein [Arabidopsis thaliana] (TAIR:AT1G68440.1); similar to IMP dehydrogenase/GMP reductase [Medicago truncatula] (GB:ABE93249.1) |  |  |  |
| 248524_s_at | At5g50570 At5g50670 | squamosa promoter-binding protein, putative |  |  | S |
| 252483_at | At3g46600 | scarecrow transcription factor family protein |  |  |  |
| 254926_at | At4g11280 | ACS6__ACS6 (1-AMINOCYCLOPROPANE-1-CARBOXYLIC ACID (ACC) SYNTHASE 6) | S, R |  | S, R |
| 264524_at | At1g10070 | ATBCAT-2; catalytic |  |  |  |
| 255331_at | At4g04330 | similar to unnamed protein product [Ostreococcus tauri] (GB:CAL56420.1); similar to Os08g0425200 [Oryza sativa (japonica cultivar-group)] (GB:NP_001061837.1) |  |  |  |
| 263881_at | At2g21820 | similar to seed maturation protein PM41, putative [Medicago truncatula] (GB:ABE86900.1) |  |  |  |
| 249353_at | At5g40420 | OLEO2_PA23__OLEO2 (OLEOSIN 2) |  |  |  |
| 266544_at | At2g35300 | late embryogenesis abundant group 1 domain-containing protein / LEA group 1 domain-containing protein |  |  |  |
| 247327_at | At5g64120 | peroxidase, putative |  |  |  |
| 246932_at | At5g25190 | ethylene-responsive element-binding protein, putative |  |  |  |
| 248964_at | At5g45340 | CYP707A3__CYP707A3 (cytochrome P450, family 707, subfamily A, polypeptide 3); oxygen binding |  |  |  |
| 249112_at | At5g43780 | APS4__APS4 |  |  | S |
| 249459_at | At5g39580 | peroxidase |  |  |  |
| 267357_at | At2g40000 | similar to unknown protein [Arabidopsis thaliana] (TAIR:AT3G55840.1); similar to putative Hs1pro-1-like receptor [Glycine max] (GB:AAG44839.1); contains InterPro domain Hs1pro-1, C-terminal; (InterPro:IPR009743); contains InterPro domain Hs1pro-1, N-terminal; (InterPro:IPR009869) | S, R | S | S, R |
| 264960_at | At1g76930 | ATEXT4_ATEXT1_ORG5__ATEXT4 (EXTENSIN 4) |  |  |  |
| 267472_at | At2g02850 | ARPN__ARPN (PLANTACYANIN); copper ion binding |  |  |  |
| 262118_at | At1g02850 | glycosyl hydrolase family 1 protein | S |  | S, R |
| 262482_at | At1g17020 | SRG1__SRG1 (SENESCENCE-RELATED GENE 1); oxidoreductase, acting on paired donors, with incorporation or reduction of molecular oxygen, 2-oxoglutarate as one donor, and incorporation of one atom each of oxygen into both donors |  |  |  |
| 262644_at | At1g62710 | BETA-VPE_BETAVPE__BETA-VPE (vacuolar processing enzyme beta); cysteine-type endopeptidase |  |  | S, R |
| 266415_at | At2g38530 | LTP2_LP2__LTP2 (LIPID TRANSFER PROTEIN 2); lipid binding |  |  |  |
| 246250_at | At4g36880 | cysteine proteinase, putative |  |  |  |
| 264079_at | At2g28490 | cupin family protein |  |  |  |
| 250468_at | At5g10120 | ethylene insensitive 3 family protein |  |  |  |
| 248448_at | At5g51190 | AP2 domain-containing transcription factor, putative |  |  |  |
| 266098_at | At2g37870 | protease inhibitor/seed storage/lipid transfer protein (LTP) family protein |  |  |  |
| 248520_at | At5g50600 At5g50700 | short-chain dehydrogenase/reductase (SDR) family protein |  |  |  |
| 253608_at | At4g30290 | ATXTH19__ATXTH19 (XYLOGLUCAN ENDOTRANSGLUCOSYLASE/HYDROLASE 19); hydrolase, acting on glycosyl bonds |  |  |  |
| 251438_s_at | At3g59930 At5g33355 | Encodes a defensin-like (DEFL) family protein. |  |  |  |
| 247543_at | At5g61600 | ethylene-responsive element-binding family protein |  |  |  |
| 265672_at | At2g31980 | cysteine proteinase inhibitor-related |  |  |  |
| 255524_at | At4g02330 | pectinesterase family protein | S |  | S |
| 251428_at | At3g60140 | DIN2_SRG2__DIN2 (DARK INDUCIBLE 2); hydrolase, hydrolyzing O-glycosyl compounds |  |  |  |
| 253915_at | At4g27280 | calcium-binding EF hand family protein | S, R |  | S |
| 263046_at | At2g05380 | GRP3S__GRP3S (GLYCINE-RICH PROTEIN 3 SHORT ISOFORM) |  |  |  |
| 262381_at | At1g72900 | disease resistance protein (TIR-NBS class), putative | S |  | S, R |
| 260551_at | At2g43510 | ATTI1__ATTI1 (ARABIDOPSIS THALIANA TRYPSIN INHIBITOR PROTEIN 1) | S, R |  | S, R |
| 260716_at | At1g48130 | ATPER1__ATPER1 (Arabidopsis thaliana 1-cysteine peroxiredoxin 1); antioxidant |  |  |  |
| 247717_at | At5g59320 | LTP3__LTP3 (LIPID TRANSFER PROTEIN 3); lipid binding |  |  |  |
| 258498_at | At3g02480 | ABA-responsive protein-related |  |  |  |
| 246099_at | At5g20230 | ATBCB_BCB__ATBCB (ARABIDOPSIS BLUE-COPPER-BINDING PROTEIN); copper ion binding |  |  |  |
| 266393_at | At2g41260 | M17__M17 |  |  |  |
| 260890_at | At1g29090 | peptidase C1A papain family protein |  |  |  |
| 247552_at | At5g60920 | COB__COB (COBRA) |  |  |  |
| 249082_at | At5g44120 | CRA1_CRU1__CRA1 (CRUCIFERINA); nutrient reservoir |  |  |  |
| 257969_at | At3g27520 | similar to hypothetical protein [Oryza sativa (japonica cultivar-group)] (GB:BAD16828.1); similar to Os02g0595300 [Oryza sativa (japonica cultivar-group)] (GB:NP_001047312.1) |  |  |  |
